# Organodichalcogenide Structure and Stability: Hierarchical Ab Initio Benchmark and DFT Performance Study

**DOI:** 10.1002/jcc.70142

**Published:** 2025-05-26

**Authors:** Steven E. Beutick, Francesco Lambertini, Trevor A. Hamlin, F. Matthias Bickelhaupt, Laura Orian

**Affiliations:** ^1^ Dipartimento di Scienze Chimiche Università Degli Studi di Padova Padova Italy; ^2^ Department of Chemistry and Pharmaceutical Sciences, Amsterdam Institute of Molecular and Life Sciences (AIMMS) Vrije Universiteit Amsterdam Amsterdam the Netherlands; ^3^ Institute of Molecules and Materials Radboud University Nijmegen the Netherlands; ^4^ Department of Chemical Sciences University of Johannesburg Johannesburg South Africa

**Keywords:** benchmark study, bond strength, coupled cluster, density functional theory, organodichalcogenides

## Abstract

We conducted a double‐hierarchical ab initio benchmark and DFT performance study of the organodichalcogenide bonding motif CH_3_Ch^1^—Ch^2^(O)_n_CH_3_ with Ch^1^, Ch^2^ = S, Se and *n* = 0, 1, 2. The organodichalcogenide model systems were optimized at ZORA‐CCSD(T)/ma‐ZORA‐def2‐TZVPP. Our ab initio benchmark involved a hierarchical series of all‐electron relativistically contracted variants of the Karlsruhe basis sets (ZORA‐def2‐SVP, ZORA‐def2‐TZVPP, ZORA‐def2‐QZVPP), both with and without diffuse functions (ma‐basis set), in conjunction with a hierarchical series of ZORA‐relativistic quantum chemical methods [HF, MP2, CCSD, and CCSD(T)]. Counterpoise correction was applied to account for the basis set superposition error (BSSE). We assessed the performance of 33 ZORA‐relativistic DFT functionals (ZORA‐[XC functional]/TZ2P//ZORA‐[XC functional]/TZ2P) against our benchmark energies and found that M06 and MN15 furnish accurate geometries and bond energies within a mean absolute error of 1.2 kcal mol^−1^ relative to our best ab initio reference data.

## Graphical abstract

We performed a double‐hierarchical ab initio benchmark study of CH_3_Ch^1^—Ch^2^(O)_n_CH_3_ (Ch^1^, Ch^2^ = S, Se; *n* = 0, 1, 2). Assessment of 33 DFT functionals showed M06 and MN15 provide accurate geometries and bond energies within 1.2 kcal mol^−1^ of our counterpoise‐correct ZORA‐CCSD(T)/ma‐ZORA‐def2‐QZVPP//ZORA‐CCSD(T)/ma‐ZORA‐def2‐TZVPP reference data.

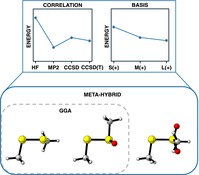

## 1 Introduction

The organodichalcogenide motif R^1^—Ch^1^—Ch^2^(O)_n_—R^2^ (Ch = chalcogen, R = organyl) has emerged as a key feature in several subfields in chemistry due to its unique chemical properties. Organodichalcogenides have found applications in catalytic biomedicine [1], biochemistry [2], fluorescent probes [3], electrochemistry [4], and this bonding motif plays an essential catalytic role in the GPx enzymatic pocket [5]. Additionally, it plays a key role in modern organic chemistry approaches due to its ability to react with nucleophiles, electrophiles, and radicals [6]. Recently, there has been a growing interest in quantifying the bonding properties of organodichalcogenide bonds across different oxidation states [6, 7]. This research is particularly valuable in redox biology and in searching for applications of organoselenides in medicinal chemistry [1, 5, 8]. In fact, selenium‐based drug candidates spread in the biological environment bonded to free cysteines or to cysteine residues of albumins. Thus, their activity implies forming and breaking Se—S bonds [5, 8]. In addition, both sulfur and selenium may be oxidized by H_2_O_2_, whose concentration is increased in oxidative stress conditions; considering different oxidation states when studying the chalcogen–chalcogen bond is therefore prominent to have a broad description of its properties [5, 8].

**SCHEME 1:**
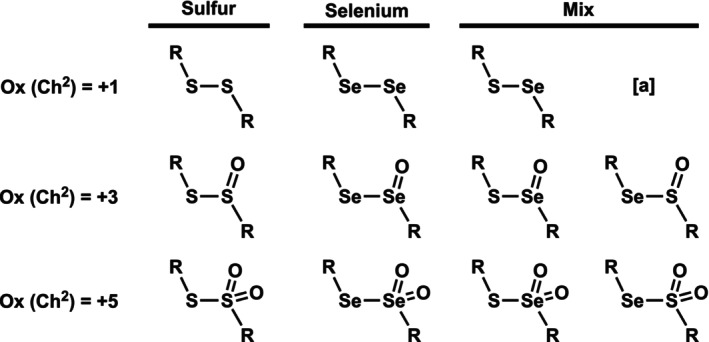
The studied organodichalcogenide model systems (CH_3_Ch^1^—Ch^2^(O)_n_CH_3_, Ch = S, Se and *n* = 0, 1, 2). The structure of H_3_CSeSCH_3_ is equal to H_3_CSSeCH_3_ and is therefore omitted.

Density functional theory (DFT) and Kohn‐Sham molecular orbital (KS‐MO) analysis are crucial for understanding the organodichalcogenide bonding mechanism. Choosing an appropriate functional and basis set combination is essential to obtain accurate energetic and structural properties for the studied system. Recently, the organodichalcogenide motif (R^1^—Ch^1^—Ch^2^—R^2^, where R = aryl) has been benchmarked against solid‐state crystal structures [9]. We build on those insights and now have performed a detailed hierarchical benchmark study based on a series of relativistic ab initio methods up to highly correlated levels for a series of organodichalcogenide model systems CH_3_Ch^1^—Ch^2^(O)_n_CH_3_, with Ch = S, Se and *n* = 0, 1, 2 (see Scheme 1). Our model systems cover the most important types of organodichalcogenide bonds. They systematically vary in the chalcogen atom (Ch^1^ = S, Se and Ch^2^ = S, Se) and number of oxide substituents (n), going from *n* = 0 to 1 to 2 (for the oxidation state going from +1 to +3 to +5).

The aim of this work is twofold. First, we present a comprehensive double‐hierarchical benchmark study of high‐level relativistic ab initio methods. The homolytic CH_3_Ch^1^—Ch^2^(O)_n_CH_3_ bond energies Δ*E* were computed using a hierarchical series of ab initio methods [HF, MP2, CCSD, and CCSD(T)] combined with a hierarchical series of increasingly flexible and polarized Gaussian‐type basis sets, including polarization functions (up to *g* basis functions) and diffuse functions (all‐electron Karlsruhe basis sets: ZORA‐def2‐SVP, ma‐ZORA‐def2‐SVP, ZORA‐def2‐TZVPP, ma‐ZORA‐def2‐TZVPP, ZORA‐def2‐QZVPP, ma‐ZORA‐def2‐QZVPP). Second, we evaluate a set of relativistic density functionals with a TZ2P basis set for predicting the organodichalcogenide bond energies Δ*E* against our best ab initio benchmark. Our study identifies M06, MN15, and MN12‐SX as well performing density functionals for exploring systems that involve the organodichalcogenide bonding motif (CH_3_Ch^1^—Ch^2^(O)_n_CH_3_). GGA functionals, including PBE and PW91, are suitable and computationally efficient alternatives if the study is limited to the organodichalcogenide bonding motif for lower oxidation states (*n* = 0, 1).

## 2 Computational Methods

### 2.1 DFT Energies and Geometries

All DFT calculations were performed with AMS2023 (an example input file is provided in the Supporting Information) [10]. The fragments were treated in a spin‐unrestricted formalism. This work focuses primarily on the homolytic dissociation pathway because heterolytic dissociation is energetically considerably less favorable in the gas phase (Table S1) [11]. All geometries and energies were computed using the TZ2P basis set for which the MOs were expanded using a large, uncontracted set of Slater‐type orbitals (STO) [12]. Scalar relativistic effects were accounted for through the zeroth order approximation (ZORA; see Table S2 in the Supporting Information) [13, 14]. The accuracy of the fit scheme (Zlm fit) [15a] and the integration grid (Becke grid) [15b] were set to VERY GOOD. The convergence criteria for the SCF and geometry optimization procedures were set to 10^−5^ Hartree. Frequency calculations were performed to characterize the nature of stationary points: energy minima had all real frequencies.

Initial conformer searches were performed using CREST to ensure global minimum structures were used [16]. The conformer search to find the lowest energy conformers was then confirmed using three DFT methods: BP86/TZ2P, BP86‐D3(BJ)/TZ2P, and M06‐2X/TZ2P. Additionally, a 360° rotational scan was performed around the C—Ch—Ch—C dihedral angle (Figure S1). The resulting structures from our thorough conformer search were used as initial structures for all geometry optimizations.

Our lowest energy conformers were reoptimized using the 33 density functionals that are grouped into local‐density approximation (LDA) functionals: VWN [17]; the generalized gradient approximation (GGA) functionals: BLYP [18], BP86 [19, 20], HTBS [21, 22], PW91 [23], mPW [23, 24], PBE [25], RPBE [22, 25], rev‐PBE [25, 26], mPBE [25, 27], HTBS [21, 25], OLYP [18, 28], OPBE [25, 28, 29]; the meta‐GGA functionals: M06‐L [30], SCAN [31], r^2^SCAN [32]; the hybrid functionals: B3LYP [18, 33], mPW1PW [23, 24], OPBE0 [29], PBE0 [34]; the meta‐hybrid functionals: M06 [30], M06‐2X [30], M06‐HF [30], MN15 [35]; and the range‐separated functionals: M11 [36], MN12‐SX [37], LCY‐PBE [38], ωB97X [39], CAM‐B3LYP [40]. The long‐range explicit D3 dispersion correction formulated by Grimme [41] combined with the suggested damping function of Becke Johnson (BJ) [42] was included for BP86‐D3(BJ), BLYP‐D3(BJ), PBE‐D3(BJ), SCAN‐D3(BJ).

### 2.2 Ab Initio Energies and Geometries

All ab initio calculations were performed using ORCA [43]. Based on our DFT conformer search (vide supra and Figure S1), the structures, initially optimized at M06‐2X/TZ2P, were reoptimized at ZORA‐CCSD(T)/ma‐ZORA‐def2‐TZVPP and were verified to be energy minima through vibrational analysis (no imaginary frequencies). Based on the optimized structures, the energies were determined for a hierarchical series of ab initio methods, that is, Hartree‐Fock theory (HF), second‐order Møller‐Plesset perturbation theory up to second order (MP2), coupled‐cluster theory with single and double excitations (CCSD), and with triple excitations treated perturbatively (CCSD(T)) combined with increasingly flexible and relativistically contracted all‐electron Karlsruhe basis sets, both with and without minimal augmentation (ma) [44a], [ZORA‐def2‐SVP, ma‐ZORA‐def2‐SVP, ZORA‐def2‐TZVPP, ma‐ZORA‐def2‐TZVPP, ZORA‐def2‐QZVPP, ma‐ZORA‐def2‐QZVPP] (Table 1) [44]. The scalar relativistic effects were included using the scalar zeroth‐order regular approximation (ZORA) [13] and the SCF convergence criteria were set to tight. All structures were visualized using CYLview [45].

**TABLE 1:** Number of relativistically contracted basis functions for the Karlsruhe basis sets without (BS#) and with (BS#+) added diffuse functions.

Basis set	Label	H	C, O	S	Se
ZORA‐def2‐SVP	BS1	2*s*1*p*	3*s*2*p*1*d*	6*s*3*p*1*d*	9*s*6*p*3*d*
ZORA‐def2‐TZVPP	BS2	3*s*2*p*1*d*	6*s*3*p*2*d*1*f*	8*s*4*p*3*d*1*f*	10*s*8*p*4*d*1*f*
ZORA‐def2‐QZVPP	BS3	4*s*3*p*2*d*1*f*	8*s*4*p*3*d*2*f*1*g*	11*s*7*p*4*d*2*f*1*g*	14*s*11*p*4*d*4*f*1*g*
ma‐ZORA‐def2‐SVP	BS1+	2*s*1*p*	4*s*3*p*1*d*	7*s*4*p*1*d*	10*s*7*p*3*d*
ma‐ZORA‐def2‐TZVPP	BS2+	3*s*2*p*1*d*	7*s*4*p*2*d*1*f*	9*s*5*p*3*d*1*f*	11*s*9*p*4*d*1*f*
ma‐ZORA‐def2‐QZVPP	BS3+	4*s*3*p*2*d*1*f*	9*s*5*p*3*d*2*f*1*g*	12*s*8*p*4*d*2*f*1*g*	15*s*12*p*4*d*4*f*1*g*

The basis set superposition error (BSSE) has been computed in each step along the double hierarchical series of ab initio methods and basis sets using the counterpoise correction (CPC) [46]. The bond energy ∆*E* is shown in Equation (1):

(1) $$\text{∆}E=E_{\mathrm{AB}}^{\mathrm{AB}}\left(\mathrm{AB}\right)-\left(E_{A}^{A}\left(A\right)-E_{B}^{B}\left(B\right)\right)$$

In the approach of counterpoise correction, the bond energy ∆*E* is corrected for the BSSE ∆*E*_BSSE_, shown in Equation (2):

(2) $$\text{∆}E_{\text{BSSE}}=\left[E_{A}^{\mathrm{AB}}\left(\mathrm{AB}\right)–E_{A}^{\mathrm{AB}}\left(A\right)+E_{B}^{\mathrm{AB}}\left(\mathrm{AB}\right)–E_{B}^{\mathrm{AB}}\left(B\right)\right]$$

The BSSE is the difference between, on one hand, the energy *E*_*A*_^*AB*^(*AB*) + *E*_*B*_^*AB*^(*AB*) of the separate fragments *A* and *B* (subscript), each computed in the geometry (superscript) and with the basis set (in parentheses) of the overall complex AB and, on the other hand, the energy *E*_*A*_^*AB*^(*A*) + *E*_*B*_^*AB*^(*B*) of the separate fragments *A* and *B* (subscript), each computed in the geometry of the overall complex *AB* (superscript) but with their own respective basis set (in parentheses). The counterpoise‐corrected bond energy (∆*E*_CPC_) is the sum of the ∆*E* and ∆*E*_BSSE_ (see Equation 3):

(3) $$\text{∆}E_{\mathrm{CPC}}=\text{∆}E+\text{∆}E_{\text{BSSE}}$$

## 3 Results and Discussion

### 3.1 Ab Initio Geometries

**FIGURE 1:**
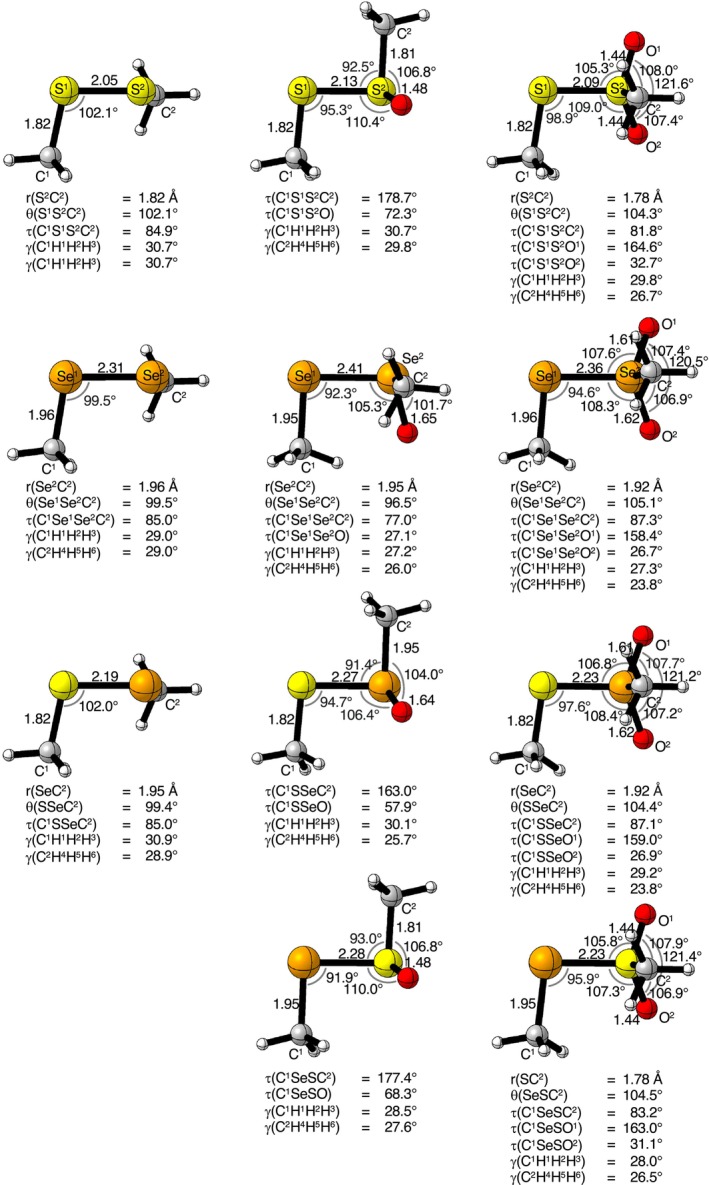
The ZORA‐CCSD(T)/BS2+ optimized geometries of the organodichalcogenide model systems. The bond distances (in Å), angles (in degrees), dihedral angle τ(C—Ch—Ch—C) (in degrees), and the pyramidalization of the methyl groups (γ(CH_x_H_y_H_z_)) are provided. The pyramidalization of the methyl groups γ(CH_x_H_y_H_z_) is defined as the deviation in the sum of the angle between the hydrogens from 180°.

First, we examine the equilibrium geometries of our organodichalcogenide model systems CH_3_Ch—Ch(O)_n_CH_3_ with Ch = S, Se, and *n* = 0, 1, 2. The geometries were optimized at ZORA‐CCSD(T)/ma‐ZORA‐def2‐TZVPP without any structural or symmetry constraints (Figure 1, for complete structural details, see Figures S2 and S3). A vibrational analysis has verified all organodichalcogenides CH_3_Ch—Ch(O)_n_CH_3_ and their radical fragments CH_3_Ch^•^ + ^•^Ch(O)_n_CH_3_ to be equilibrium geometries (no imaginary frequencies).

The central chalcogen–chalcogen bond (r(Ch—Ch)) in the organodichalcogenides becomes longer as the size of the chalcogen atoms increases from S to Se. Compared to sulfur‐containing organodichalcogenides (S—S(O)_n_), selenium (Se—Se(O)_n_) and mixed organodichalcogenides (S—Se(O)_n_ and Se—S(O)_n_) feature longer central chalcogen–chalcogen bond distances by on average 0.27 and 0.25 Å, respectively. Furthermore, as the oxidation state increases from +1 to +3, the chalcogen–chalcogen bond distance increases significantly by approximately 0.10 Å and then slightly decreases by around 0.05 Å on going from the oxidation state +3 to +5.

As seen in Figure 1, the two methyl groups are nearly perpendicular around the dihedral angle with τ(C—Ch—Ch—C) ranging from 80° to 100°. However, in organodichalcogenides with an oxidation state of +3, the equilibrium geometry adopts an *anti*‐periplanar conformation, with the oxide positioned out of the plane. For SeSeO, there is sufficient space for the oxygen to rotate in the *syn*‐periplanar position to form an internal hydrogen bond. This internal hydrogen bond is present in all organodichalcogenides where the oxidation state is +5.

### 3.2 Ab Initio Ch—Ch Bond Energies

**FIGURE 2:**
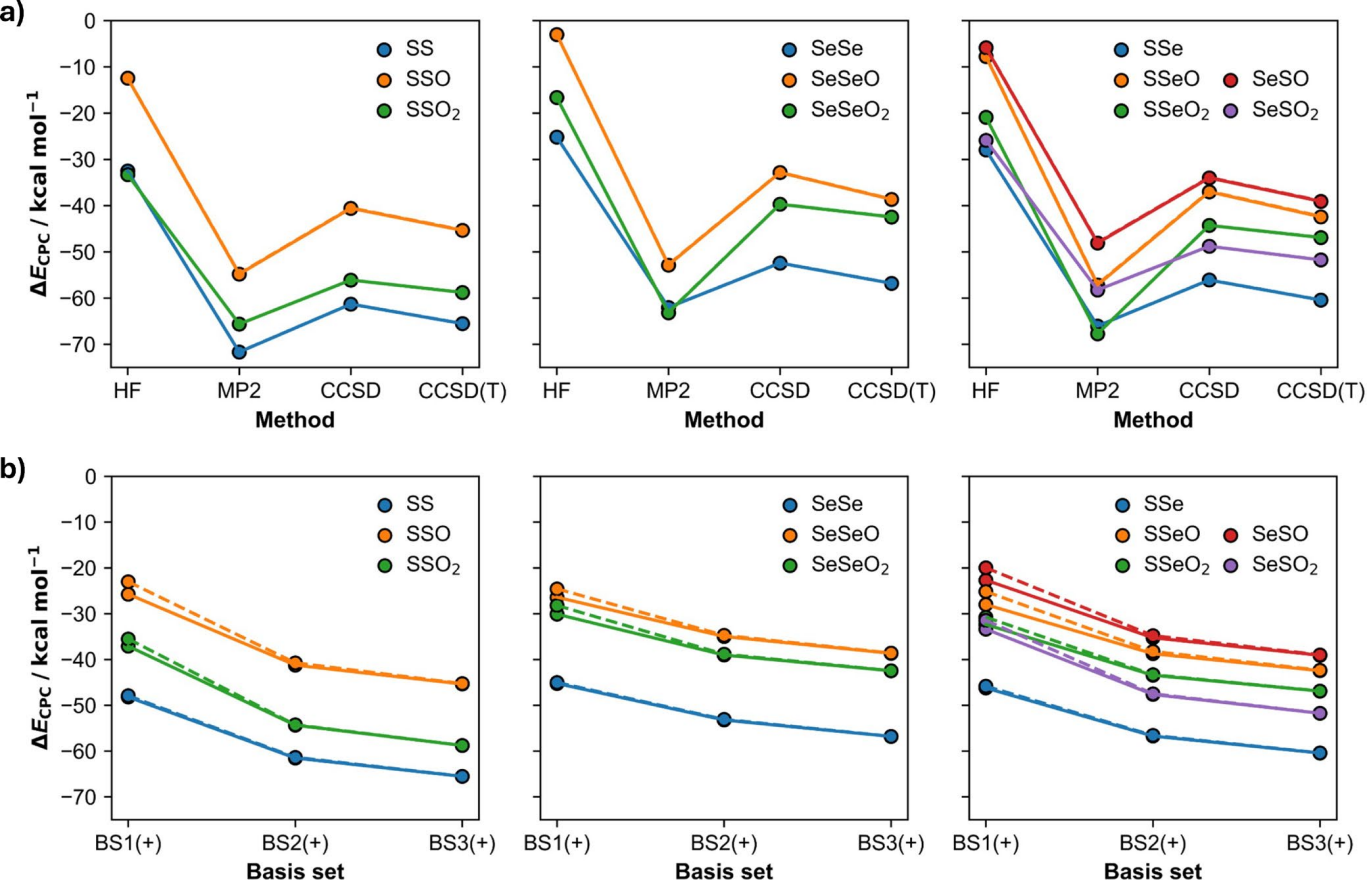
Counterpoise‐corrected bond energies Δ*E*_CPC_ (a) along hierarchical series of ab initio methods in combination with BS3+; and (b) along BS1 to BS3 (dashed lines) and BS1+ to BS3+ (solid lines) basis sets. All energies were computed at ZORA‐Method/BS#+//ZORA‐CCSD(T)/BS2+.

The counterpoise‐corrected (Δ*E*_CPC_) and uncorrected (Δ*E*) bond energies for various organodichalcogenide bonds as a function of the hierarchical series of ab initio methods and Gaussian basis sets are provided in Figure 2. The geometries were optimized and confirmed to be local minima (no imaginary frequencies) at ZORA‐CCSD(T)/ma‐ZORA‐def2‐TZVPP (ZORA‐CCSD(T)/BS2+). For the fragments (CH_3_Ch^•^), the spin contamination should be considered because unrestricted formalism is applied. The restricted Hartree‐Fock (RHF) method constrains alpha‐spin and beta‐spin orbitals to be identical, producing a pure doublet state with a spin‐squared expectation value ⟨*S* ^2^⟩ of 0.75 (*s*(*s* + 1) for *s* = 1/2). The unrestricted Hartree‐Fock (UHF) method allows these orbitals to differ, which lowers the energy but introduces spin contamination by states of higher spin multiplicity, causing the ⟨*S* ^2^⟩ value to exceed 0.75. Generally, the error associated with the spin‐contaminated wavefunction is considered negligible if the spin‐squared expectation value falls within 10% of the spin‐pure ⟨*S* ^2^⟩ value [47]. This is the case for our radical fragments (Tables S3 and S4). The results of our ab initio computations are summarized in Tables 2, 3, and S5–S8.

**TABLE 2:** Homolytic Ch^1^—Ch^2^ bond energies (in kcal mol^−1^) of S—S and Se—Se organodichalcogenide bonds with (∆*E*_CPC_) and without (∆*E*) counterpoise corrections included.^a^

Method	Basis set	SS		SSO		SSO_2_		SeSe		SeSeO		SeSeO_2_	
		∆*E*_CPC_	∆*E*	∆*E*_CPC_	∆*E*	∆*E*_CPC_	∆*E*	∆*E*_CPC_	∆*E*	∆*E*_CPC_	∆*E*	∆*E*_CPC_	∆*E*
HF	BS1	−26.5	−29.4	−0.7	−4.8	−22.5	−28.1	−23.1	−24.6	2.0	−0.6	−11.6	−15.1
	BS2	−32.2	−32.7	−11.8	−12.4	−32.9	−33.8	−24.7	−25.3	−2.3	−3.1	−16.2	−17.1
	BS3	−32.5	−32.6	−12.4	−12.5	−33.3	−33.5	−25.2	−25.3	−3.0	−3.1	−16.6	−16.8
MP2	BS1	−52.3	−59.7	−31.1	−39.4	−41.1	−51.3	−49.0	−53.4	−36.5	−43.1	−48.0	−55.1
	BS2	−67.7	−69.6	−50.2	−52.5	−61.3	−64.3	−58.0	−61.1	−48.6	−52.3	−59.4	−63.3
	BS3	−71.7	−72.5	−54.7	−55.8	−65.6	−66.9	−62.0	−63.9	−52.8	−54.8	−63.1	−65.2
CCSD	BS1	−45.4	−53.4	−19.8	−28.3	−34.2	−44.8	−42.3	−47.1	−20.4	−27.1	−26.7	−33.8
	BS2	−57.7	−59.5	−36.5	−38.7	−52.0	−54.8	−49.4	−52.4	−29.5	−32.9	−36.5	−40.1
	BS3	−61.3	−61.9	−40.5	−41.3	−56.1	−57.1	−52.4	−53.9	−32.8	−34.5	−39.7	−41.5
CCSD(T)	BS1	−47.9	−56.3	−23.0	−32.0	−35.5	−46.6	−45.0	−50.1	−24.6	−31.7	−28.2	−35.8
	BS2	−61.4	−63.5	−40.7	−43.2	−54.3	−57.4	−53.1	−56.2	−34.7	−38.4	−38.8	−42.7
	BS3	−65.5	−66.2	−45.3	−46.2	−58.8	−59.9	−56.8	−58.4	−38.6	−40.4	−42.4	−44.4
HF	BS1+	−26.5	−28.2	−3.5	−6.1	−23.7	−27.3	−23.0	−24.0	−0.3	−2.0	−13.8	−15.8
	BS2+	−32.2	−32.5	−12.1	−12.4	−32.8	−33.3	−24.7	−25.2	−2.5	−2.9	−16.2	−16.7
	BS3+	−32.5	−32.6	−12.5	−12.5	−33.3	−33.4	−25.2	−25.2	−3.0	−3.1	−16.6	−16.7
MP2	BS1+	−52.9	−59.4	−34.2	−41.3	−42.8	−51.5	−49.4	−53.5	−39.1	−44.4	−50.2	−56.0
	BS2+	−67.8	−69.7	−50.7	−52.8	−61.4	−63.8	−58.1	−61.2	−49.0	−52.3	−59.6	−63.2
	BS3+	−71.7	−72.5	−54.8	−55.8	−65.6	−66.8	−62.0	−63.9	−52.9	−54.9	−63.2	−65.2
CCSD	BS1+	−45.8	−52.7	−22.5	−29.9	−35.8	−44.8	−42.5	−46.9	−22.3	−27.7	−28.5	−34.4
	BS2+	−57.8	−59.5	−36.9	−38.8	−52.1	−54.4	−49.5	−52.4	−29.7	−32.7	−36.7	−39.9
	BS3+	−61.3	−61.9	−40.6	−41.3	−56.1	−57.0	−52.4	−54.0	−32.9	−34.5	−39.7	−41.4
CCSD(T)	BS1+	−48.2	−55.7	−25.7	−33.7	−37.1	−46.8	−45.2	−50.1	−26.4	−32.4	−30.1	−36.6
	BS2+	−61.5	−63.5	−41.2	−43.4	−54.4	−56.9	−53.2	−56.3	−35.0	−38.3	−39.0	−42.5
	BS3+	−65.5	−66.2	−45.4	−46.2	−58.8	−59.8	−56.8	−58.5	−38.7	−40.4	−42.5	−44.3

^a^ Computed at ZORA‐Method/BS#//ZORA‐CCSD(T)/BS2+. See Figure 2a for the graphical representation of the counterpoise‐corrected energies (∆*E*_CPC_) as function of the ab initio method and Figure 2b as a function of the basis sets.

**TABLE 3:** Homolytic Ch^1^—Ch^2^ bond energies (in kcal mol^−1^) of mixed S—Se organodichalcogenide model systems with (∆*E*_CPC_) and without (∆*E*) counterpoise corrections.^a^

Method	Basis set	SSe		SSeO		SSeO_2_		SeSO		SeSO_2_	
		∆*E*_CPC_	∆*E*	∆*E*_CPC_	∆*E*	∆*E*_CPC_	∆*E*	∆*E*_CPC_	∆*E*	∆*E*_CPC_	∆*E*
HF	BS1	−24.1	−26.3	0.0	−3.3	−14.6	−18.7	3.9	0.6	−17.2	−22.1
	BS2	−27.6	−28.2	−7.0	−7.6	−20.6	−21.5	−5.2	−5.9	−25.4	−26.3
	BS3	−28.0	−28.1	−7.7	−7.8	−20.9	−21.1	−5.8	−5.9	−25.9	−26.1
MP2	BS1	−49.9	−55.8	−37.4	−45.0	−50.7	−59.3	−27.7	−34.6	−36.6	−45.3
	BS2	−62.1	−64.8	−53.0	−55.9	−64.4	−67.8	−43.5	−46.4	−53.8	−57.5
	BS3	−66.0	−67.5	−57.0	−58.6	−67.7	−69.5	−48.0	−49.6	−58.2	−60.1
CCSD	BS1	−43.1	−49.6	−21.2	−29.1	−29.2	−38.0	−16.4	−23.4	−29.9	−38.8
	BS2	−52.9	−55.3	−33.3	−36.0	−41.0	−44.2	−30.3	−33.1	−45.1	−48.4
	BS3	−56.1	−57.2	−36.9	−38.2	−44.3	−45.7	−33.9	−35.2	−48.8	−50.3
CCSD(T)	BS1	−45.8	−52.6	−25.2	−33.3	−30.8	−39.8	−20.0	−27.4	−31.5	−40.8
	BS2	−56.6	−59.3	−38.2	−41.2	−43.3	−46.8	−34.8	−37.8	−47.5	−51.2
	BS3	−60.4	−61.7	−42.3	−43.7	−46.9	−48.5	−39.0	−40.4	−51.7	−53.4
HF	BS1+	−24.2	−25.6	−3.2	−5.3	−16.5	−19.1	1.1	−1.1	−18.7	−22.0
	BS2+	−27.6	−28.0	−7.3	−7.6	−20.5	−21.0	−5.5	−5.9	−25.4	−25.9
	BS3+	−28.0	−28.0	−7.8	−7.8	−20.9	−21.0	−5.9	−5.9	−25.9	−26.0
MP2	BS1+	−50.6	−55.9	−41.2	−47.5	−52.3	−60.2	−30.6	−36.5	−38.6	−45.9
	BS2+	−62.3	−64.8	−53.6	−56.3	−64.5	−67.7	−44.0	−46.7	−54.0	−57.2
	BS3+	−66.0	−67.5	−57.2	−58.7	−67.7	−69.4	−48.1	−49.7	−58.3	−60.0
CCSD	BS1+	−43.6	−49.4	−24.2	−30.7	−30.9	−38.5	−19.1	−25.1	−31.8	−39.3
	BS2+	−53.0	−55.3	−33.7	−36.2	−41.1	−43.8	−30.8	−33.2	−45.2	−48.1
	BS3+	−56.1	−57.2	−37.0	−38.2	−44.3	−45.6	−34.0	−35.3	−48.8	−50.3
CCSD(T)	BS1+	−46.2	−52.5	−28.0	−35.1	−32.4	−40.6	−22.7	−29.2	−33.4	−41.5
	BS2+	−56.7	−59.3	−38.7	−41.5	−43.4	−46.5	−35.3	−38.0	−47.6	−50.9
	BS3+	−60.4	−61.7	−42.5	−43.8	−46.9	−48.4	−39.1	−40.5	−51.8	−53.4

^a^ Computed at ZORA‐Method/BS#//ZORA‐CCSD(T)/BS2+. See Figure 2a for the graphical representation of the counterpoise‐corrected energies (∆*E*_CPC_) as function of the ab initio method and Figure 2b as a function of the basis sets.

Our double hierarchical benchmark shows that the energies from our counterpoise‐corrected ZORA‐CCSD(T)/BS3+//ZORA‐CCSD(T)/BS2+ level converged within 3.5–4.4 and 2.6–5.8 kcal mol^−1^ with respect to the basis set and ab initio method, respectively. The trends in organodichalcogenide bond energies reflect those in the bond distance (vide supra), that is, the bonds in oxidation state +1 are strongest for CH_3_S—SCH_3_ (∆*E*_CPC_ = −65.5 kcal mol^−1^), weakest for CH_3_Se—Se CH_3_ (∆*E*_CPC_ = −56.8 kcal mol^−1^), and mixed organodichalcogenides fall in between (Tables 2 and 3). Along CH_3_S—SCH_3_, CH_3_S—S(O)CH_3_ and CH_3_S—S(O)_2_CH_3_ (i.e., oxidation states +1, +3, +5), the S—S bond first weakens by ca 20 kcal mol^−1^ and then strengthens by ca 15 kcal mol^−1^, as ∆*E*_CPC_ varies from −65.5 to −45.4 to −58.8 kcal mol^−1^.

Figure 2a shows the counterpoise‐corrected bond energies Δ*E*_CPC_ of the organodichalcogenides as a function of the ab initio method, along with BS3 (dashed lines) and BS3+ (solid lines). The HF method, which neglects Coulomb correlation, leads to a significant underestimation of bond strengths. MP2, on the other hand, overestimates the bond energies, especially for the systems containing the SeO_2_CH_3_ fragment. HF/BS3+ and MP2/BS3+ do not furnish the same trend in bond strengths as our most reliable data (ZORA‐CCSD(T)/BS3+), whereas ZORA‐CCSD/BS3+ is consistent. The counterpoise‐corrected ZORA‐CCSD(T)/BS3+//ZORA‐CCSD(T)/BS2+ bond energies converge within 2.6–5.8 kcal mol^−1^ with respect to the series of ab initio methods. There is no significant change in bond energies upon moving from BS3 to BS3+.

With respect to the basis set, the counterpoise‐corrected ZORA‐CCSD(T)/BS3+//ZORA‐CCSD(T)/BS2+ bond energies converge within 3.5–4.4 kcal mol^−1^. In Figure 2b, it shows how the ∆*E*_CPC_ values at ZORA‐CCSD(T) converge as a function of an increasingly flexible and polarized basis set. Here, we see that trends along the different bonds become consistent from a basis set BS2 or larger. Adding diffuse *s* and *p* basis functions to either BS1, BS2, or BS3 has a negligible effect on ∆*E*_CPC_ values. Only for the oxidized organodichalcogenides, and only for BS1, is there a marginal improvement.

**FIGURE 3:**
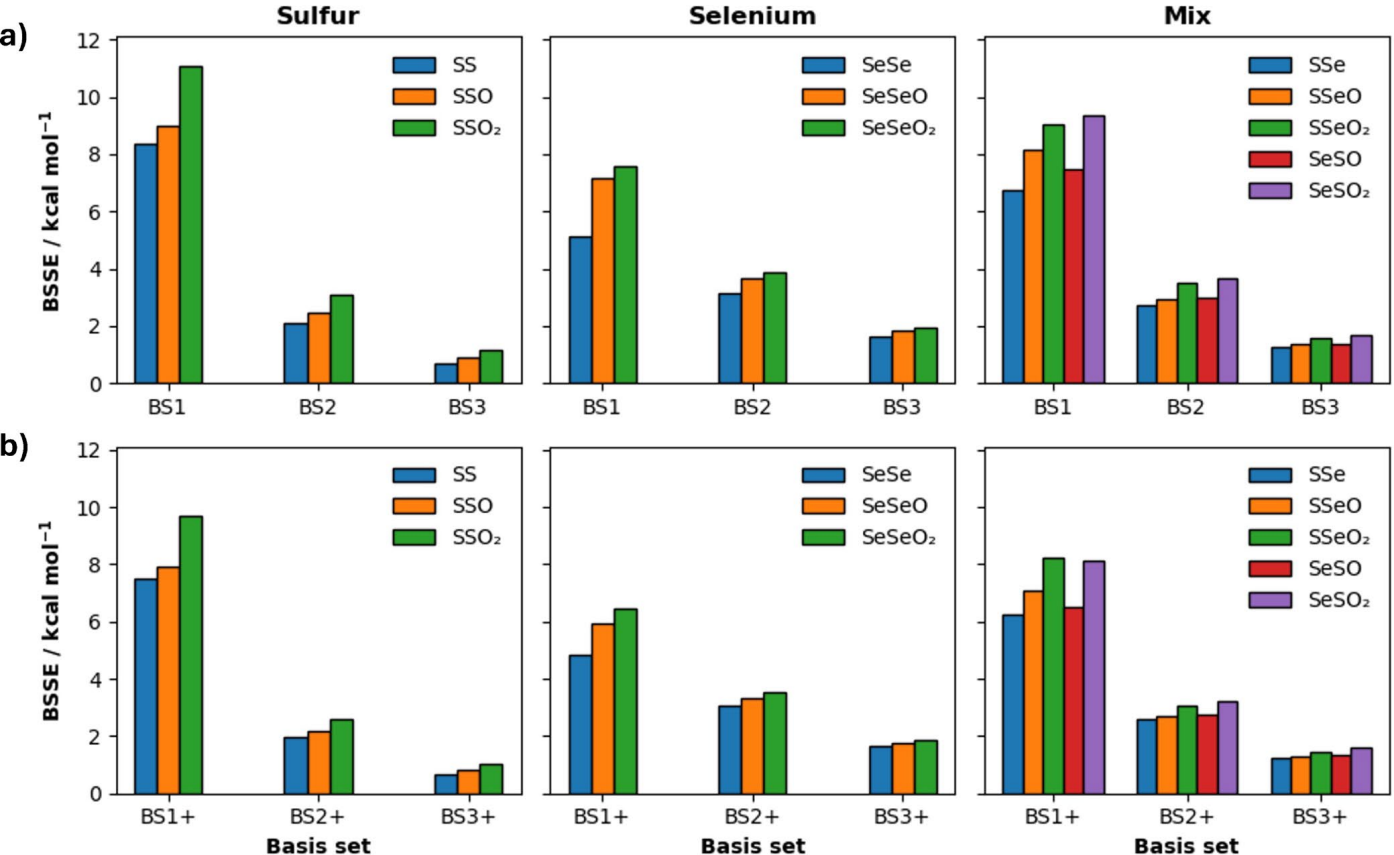
Basis set superposition error (BSSE) at ZORA‐CCSD(T)/BS# for the organodichalcogenide bonding motif along (a) BS1 to BS3 and (b) BS1+ to BS3+ basis sets.

The BSSE values at ZORA‐CCSD(T)/BS# are provided in Figure 3 (for full information on all BSSE values, see Table S6). The organodichalcogenide systems are divided into three groups, containing: only sulfur (SS, SSO, SSO_2_), only selenium (SeSe, SeSeO, SeSeO_2_), and both sulfur and selenium (SSe, SSeO, SSeO_2_, SeSO, SeSO_2_). The BSSEs increase upon going from oxidation state +1 to +3 to +5. The BSSE values decrease on going from sulfur to selenium for BS1+ and increase for larger basis sets (BS2+ and BS3+). The effect of adding diffuse basis functions on the BSSEs is limited to 0.1 kcal mol^−1^ for the BS3+ basis set (0.5 kcal mol^−1^ for BS2+ and 1.4 kcal mol^−1^ for BS1+). The BSSE is greatly reduced from 4.8–9.7 kcal mol^−1^ at ZORA‐CCSD(T)/BS1+ to 0.7–1.9 kcal mol^−1^ at ZORA‐CCSD(T)/BS3+ as the Gaussian basis set becomes more flexible.

In conclusion, our double‐hierarchical benchmark study yields counterpoise‐corrected ZORA‐CCSD(T)/BS3+//ZORA‐CCSD(T)/BS2+ bond energies that have converged within 3.5–4.4 kcal mol^−1^ with respect to extending the basis set and within 2.6–5.8 kcal mol^−1^ with respect to the level of correlation treatment in the ab initio method. In the following, our best estimates of the counterpoise‐corrected bond energies and geometries, at ZORA‐CCSD(T)/BS3+//ZORA‐CCSD(T)/BS2+, will be used to evaluate the performance of a suite of DFT approximations.

### 3.3 DFT Geometries

We have computed the equilibrium geometries of the model organodichalcogenides and the corresponding bond energies, enthalpies, and Gibbs free energies for various DFT functionals in combination with the all‐electron TZ2P basis set and ZORA for the relativistic effects. The absolute differences in bond dissociation energies computed using the TZ2P relative to the QZ4P basis set at ZORA‐[XC functional]/[basis set] for BP86, BP86‐D3(BJ), and M06‐2X amount to 0.1–1.6 kcal mol^−1^ (Table S9). These variations did not cause any significant numerical differences or alter the trends. Therefore, the computationally more efficient TZ2P basis set was selected for the complete DFT performance study, as shown in Table S10. We evaluate the quality of the DFT‐optimized geometries by comparing them with our ab initio ZORA‐CCSD(T)/BS2+ optimized structures utilizing the root‐mean‐square deviation of atomic positions (RMSD) [48], the chalcogen–chalcogen bond distance (r(Ch—Ch)), and dihedral angle between the two methyl groups (τ(C—Ch—Ch—C)). Additionally, the performance of the density functionals is discussed based on the computed ZORA‐[XC functional]/TZ2P// ZORA‐[XC functional]/TZ2P bond energies (Δ*E*_CPC_) compared to our ab initio energies computed at ZORA‐CCSD(T)/BS3+//ZORA‐CCSD(T)/BS2+.

**FIGURE 4:**
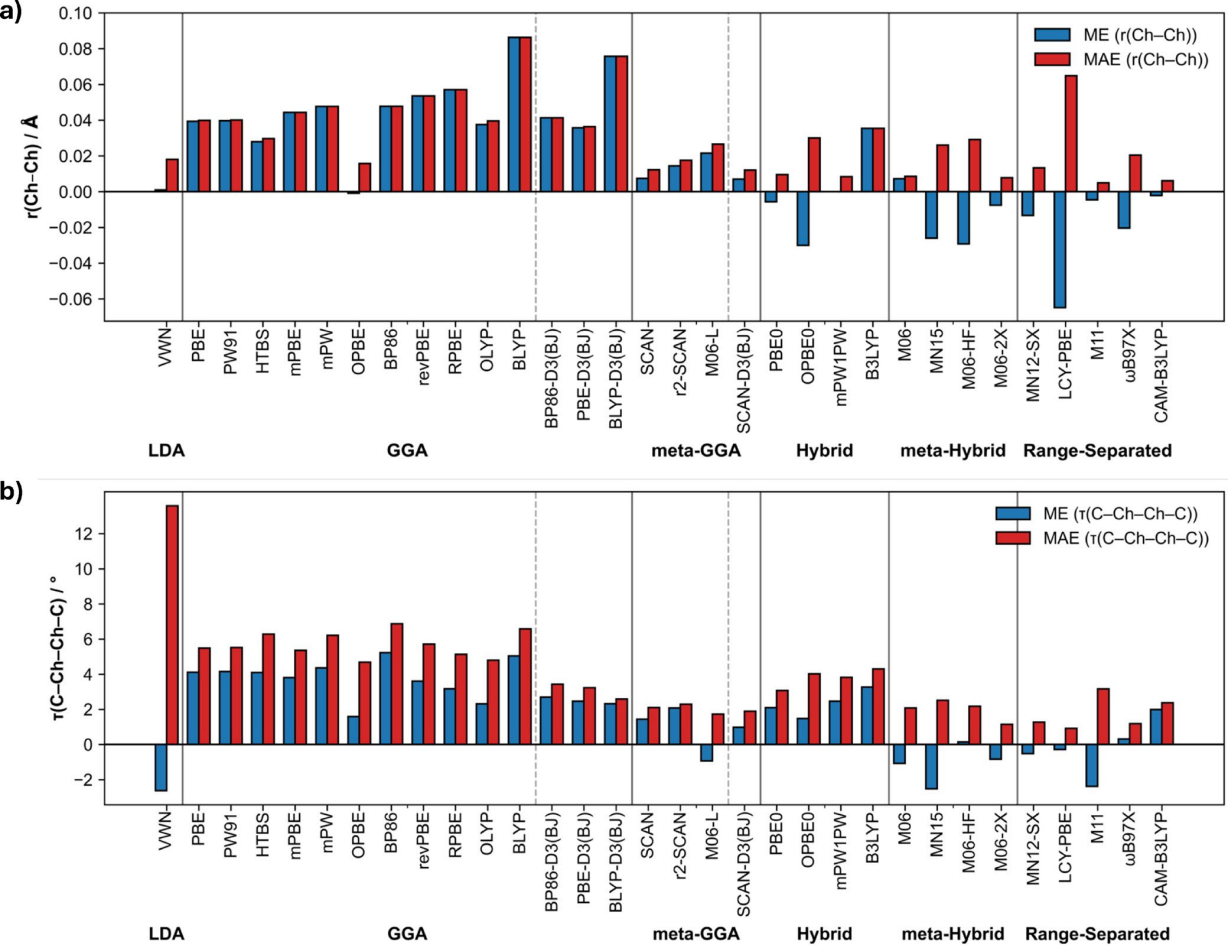
Mean error (ME) and mean absolute error (MAE) in (a) the chalcogen–chalcogen bond distance r(Ch—Ch) and (b) the dihedral angle τ(C—Ch—Ch—C) between the two methyl groups for the equilibrium geometry of our organodichalcogenide model systems computed at ZORA‐[XC functional]/TZ2P//ZORA‐[XC functional]/TZ2P compared to reference geometries computed at ZORA‐CCSD(T)/BS2+. The XC functionals were ordered based on MAE in bond energy (not based on MAE in geometry parameters) within their respective method of XC approximation.

The DFT‐optimized geometries were compared to those obtained through geometry optimization at ZORA‐CCSD(T)/BS2+. For the DFT‐geometry performance study, we isolated two significant geometric metrics, namely the error in the chalcogen–chalcogen bond distance r(Ch—Ch) (Figure 4a) and the error in the dihedral angle between the two methyl groups τ(C—Ch—Ch—C) (Figure 4b). Most trends are accurately captured; however, for the oxidation state +5, GGAs fail to fully reproduce the key trends in chalcogen–chalcogen bond distances (see Tables S11 and S12). Additionally, the RMSD, as implemented by Kabsch, was used to determine the likeness of the two sets of cartesian coordinates for both the organodichalcogenide model systems and fragments (Figure S4) [48].

From the overall DFT performance assessment, it is evident that PBE0, M06, and MN12‐SX excel in describing the geometric properties of organodichalcogenide bonds considered in this study. These functionals successfully reproduce the trends in bond distances r(Ch—Ch) observed in geometry‐optimized structures at ZORA‐CCSD(T)/BS2+ (Tables S11 and S12). Furthermore, the deviations in structural parameters are minimal. The root‐mean‐square deviation (RMSD) of atomic positions is 0.07, 0.04, and 0.03 Å for PBE0, M06, and MN12‐SX, respectively. The mean absolute error (MAE) in bond distances r(Ch—Ch) is remarkably low at 0.01 Å for all three functionals. Similarly, the MAE in dihedral angle τ(C—Ch—Ch—C) is 3.1°, 2.1°, and 1.3° for PBE0, M06, and MN12‐SX, respectively. These results highlight the reliability of these functionals in accurately capturing the structural characteristics of organodichalcogenides.

### 3.4 DFT Ch—Ch Bond Energies

We have systematically computed the homolytic Ch—Ch bond energies of organodichalcogenide model systems using a range of density‐functional approximations, from LDA and GGA to meta‐GGA, hybrid, meta‐hybrid, and range‐separated functionals, in conjunction with the TZ2P basis set (ZORA‐[XC functional]/TZ2P//ZORA‐[XC functional]/TZ2P) (Table S10). In doing so, we have established oxidation‐dependent trends across different chalcogen combinations within the same oxidation state.

Our DFT calculations generally capture the expected trends in homolytic Ch—Ch bond strengths when benchmarked against counterpoise‐corrected ZORA‐CCSD(T)/BS3+//ZORA‐CCSD(T)/BS2+. However, GGA functionals fail to reproduce the trend across the three oxidation states of selenium in the studied organodichalcogenides, specifically in CH_3_SeSe(O)_n_CH_3_ and CH_3_SSe(O)_n_CH_3_ (*n* = 0, 1, 2; Tables S13 and S14). In particular, for CH_3_SeSe(O)_2_CH_3_, GGA functionals underestimate bond strengths by approximately 10 kcal mol^−1^ (∼20%) compared to reference values (Δ*E*_CPC_ = −44.3 and −48.4 kcal mol^−1^). These findings indicate that GGA functionals are unreliable for high‐oxidation‐state selenium‐containing organodichalcogenides.

**FIGURE 5:**
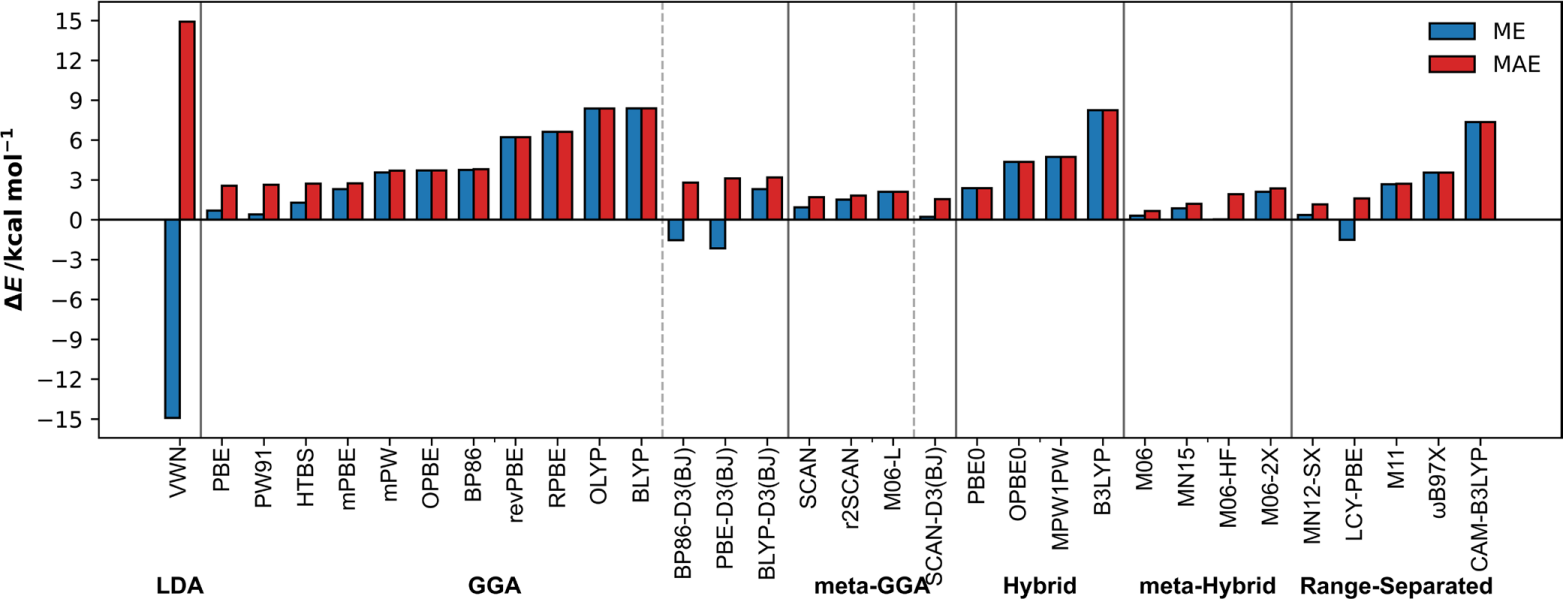
Mean error (ME) and mean absolute error (MAE) of ZORA‐[XC functional]/TZ2P//ZORA‐[XC functional]/TZ2P bond energies (∆*E*) relative to counterpoise‐corrected ZORA‐CCSD(T)/BS3+//ZORA‐CCSD(T)/BS2+ reference energies (∆*E*_CPC_). XC functionals are ordered by MAE within their respective approximation method.

To assess functional performance, we compared the mean error (ME) and MAE of computed bond energies relative to benchmark counterpoise‐corrected ZORA‐CCSD(T)/BS3+//ZORA‐CCSD(T)/BS2+ values (Δ*E*_CPC_; Figure 5, Tables S15 and S16). DFT bond energies predominantly exhibit underbinding, as indicated by the positive ME values, with few exceptions. For example, bond energies calculated with ZORA‐BP86/TZ2P//ZORA‐BP86/TZ2P have an ME of 3.75 kcal mol^−1^ and an MAE of 3.80 kcal mol^−1^, consistently underestimating bond strengths. Exceptions include the LDA functional VWN, which exhibits an average overbinding of 13.6 kcal mol^−1^. Additionally, dispersion‐corrected GGAs tend to overbind, as Grimme's D3 correction paired with the BJ damping strengthens bond energies, reducing underbinding (positive ME) and improving MAE alignment with reference values. For the SCAN functional, this effect is minor, reducing MAE by only 0.1 kcal mol^−1^, likely due to SCAN's intrinsic ability to account for intermediate‐range van der Waals interactions [31].

Despite general underbinding, several functionals demonstrate strong performance in predicting both trends and absolute bond energies. The Minnesota functionals (M06, MN15, MN12‐SX) show excellent accuracy, with MAEs of 0.7, 1.2, and 1.2 kcal mol^−1^ and largest absolute deviations (LADs) of 1.9, 3.0, and 2.7 kcal mol^−1^, respectively (Table S17). Computationally efficient alternatives, such as meta‐GGA functionals SCAN, r2SCAN, and SCAN‐D3(BJ), also perform well, with MAEs of 1.7, 1.8, and 1.6 kcal mol^−1^ and LADs of 4.4, 4.9, and 3.6 kcal mol^−1^, respectively.

**FIGURE 6:**
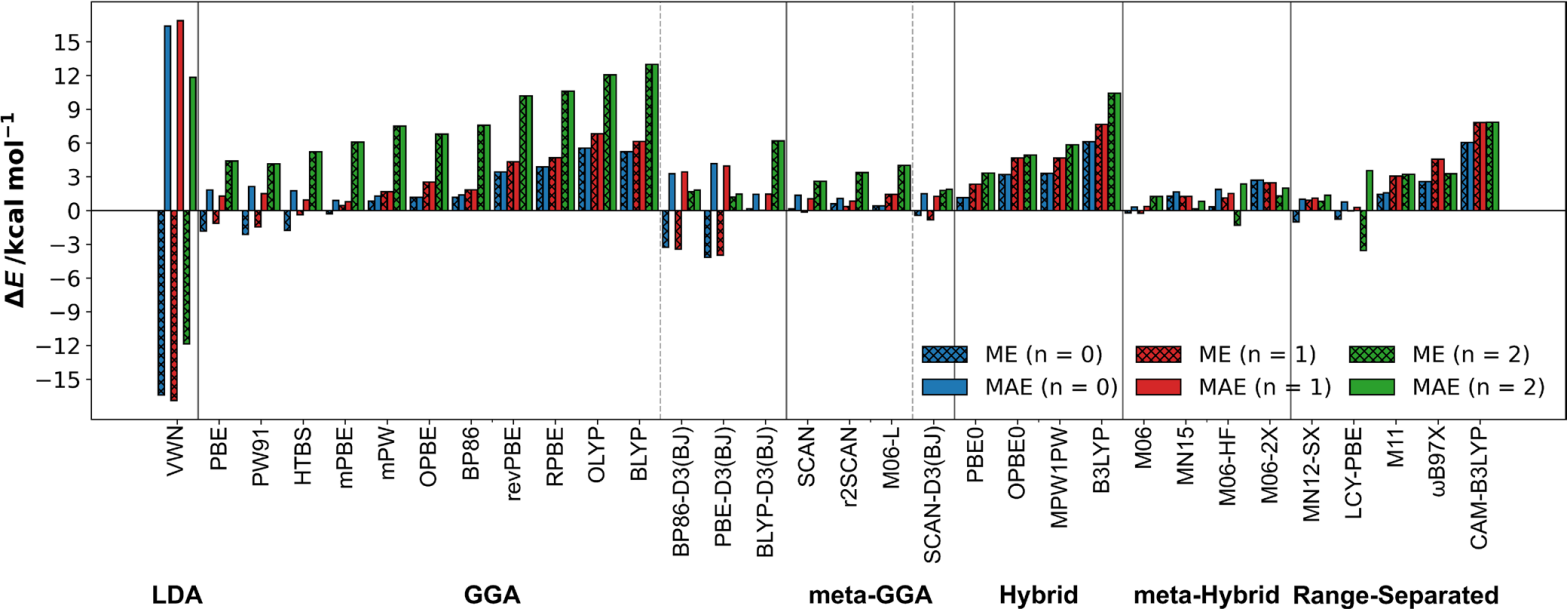
Mean error (ME) and mean absolute error (MAE) of ZORA‐[XC functional]/TZ2P//ZORA‐[XC functional]/TZ2P bond energies (∆*E*) relative to counterpoise‐corrected ZORA‐CCSD(T)/BS3+//ZORA‐CCSD(T)/BS2+ reference energies (∆*E*_CPC_) for each oxidation state in this study. XC functionals are ordered by MAE within their respective approximation method.

A key finding is that the degree of DFT underestimation in homolytic bond energy (Δ*E*) increases with the oxidation state of the chalcogen atom Ch^2^ in CH_3_Ch^1^—Ch^2^(O)_n_CH_3_, progressing from +1 to +3 to +5 (Figure 6). To better understand these deviations, we classified the organodichalcogenides into two groups: (1) by oxidation state (+1, +3, +5) and (2) by chalcogen composition (S—S, Se—Se, or mixed S—Se; Table S18). While errors in bond energy did not exhibit a strong correlation with specific chalcogen pairs, oxidation state significantly influenced error magnitude. For most GGA functionals, bond energy errors in oxidation states +1 and +3 remained within 6 kcal mol^−1^, whereas for oxidation state +5, errors often exceeded 10 kcal mol^−1^. Among GGAs, mPBE, mPW, and BLYP‐D3(BJ) performed best for lower oxidation states, with MAEs of 0.8, 1.5, and 1.5 kcal mol^−1^, respectively, in CH_3_Ch^1^—Ch^2^(O)_n_CH_3_ systems where *n* = 0 or 1. These results provide valuable insights into the reliability of different functionals for organodichalcogenide bond energy predictions. While GGA functionals struggle with high‐oxidation‐state selenium compounds, M06, MN15, MN12‐SX, and SCAN‐D3(BJ) offer significantly better accuracy. We recommend mPBE, mPW, and BLYP‐D3(BJ) for computationally efficient calculations in systems with oxidation states up to +3, whereas hybrid or Minnesota functionals are preferable for high‐oxidation‐state compounds.

## 4 Conclusions

We conducted a comprehensive double‐hierarchical relativistic ab initio benchmark study to determine the geometries and homolytic chalcogen–chalcogen bond energies of organodichalcogenide model systems of the form CH_3_Ch^1^—Ch^2^(O)_n_CH_3_, where Ch^1^, Ch^2^ = S, Se, and *n* = 0, 1, 2. Using the resulting ab initio reference data, we assessed the performance of 33 relativistic density functionals in describing these systems. The homolytic Ch—Ch bond energies showed convergence within a range of 2.6 to 5.8 kcal mol^−1^ regarding the level of correlation treatment [spanning HF, MP2, CCSD, and CCSD(T)]. Similarly, convergence with respect to basis set flexibility and polarization was achieved within 3.5 to 4.4 kcal mol^−1^. Diffuse functions were found to have a negligible impact on both accuracy and convergence for bond energies.

Employing our most accurate computational approach, that is, counter‐poise corrected ZORA‐CCSD(T)/ma‐ZORA‐def2‐QZVPP//ZORA‐CCSD(T)/ma‐ZORA‐def2‐TZVPP, we identified systematic trends in homolytic Ch^1^—Ch^2^ bond energies. The bond energy decreases with increasing selenium content, following the trend CH_3_S—S(O)_n_CH_3_ > CH_3_S—Se(O)_n_CH_3_ ~ CH_3_Se—S(O)_n_CH_3_ > CH_3_Se—Se(O)_n_CH_3_. Furthermore, the bond strength exhibits a V‐shaped dependency on the number of oxygen in the organodichalcogenide bond: it decreases from *n* = 0 to 1 and subsequently increases from *n* = 1 to 2.

The performance of the 33 ZORA‐relativistic density functionals, in conjunction with the Slater‐type TZ2P basis set, was systematically evaluated for their ability to describe the bonding energies and structural properties of organodichalcogenide model systems. For systems in lower oxidation states, such as CH_3_Ch—ChCH_3_ (+1) and CH_3_Ch—ChOCH_3_ (+3), the mPBE, mPW, and BLYP‐D3(BJ) functionals emerged as efficient and accurate methods for modeling organodichalcogenide bonding. In contrast, systems in higher oxidation states require additional considerations to achieve reliable results. Among all functionals tested, the meta‐hybrid functionals M06 and MN15, as well as the range‐separated functional MN12‐SX, demonstrated the best overall performance across the full range of organodichalcogenides studied. These three functionals consistently provided accurate results across all oxidation states, with MAEs limited to 0.7, 1.2, and 1.2 kcal mol^−1^, respectively, compared to high‐level ab initio benchmarks. These findings offer robust methods for finding novel insights and use for the organodichalcogenide bond, encompassing different oxidation states for the chalcogen nuclei, a relevant topic in chemistry and closely related fields, that is, redox biology and medicinal chemistry.

## Conflicts of Interest

The authors declare no conflicts of interest.

## Supporting information


**Data S1.** jcc70142‐sup‐0001‐Supinfo.

## Data Availability

The data to support the findings of this are available in the Supporting Information of this article. The cartesian coordinates and electronic energies are given in Tables S19 and S20.
