# Supplementary material for: Organodichalcogenide Structure and Stability: Hierarchical Ab Initio Benchmark and DFT Performance Study
Source: J Comput Chem. 2025 May 26;46(14):e70142. doi: 10.1002/jcc.70142 (PMC12105763; doi:10.1002/jcc.70142)
Supplement: Supplementary file 1 — Data S1. jcc70142‐sup‐0001‐Supinfo. [file JCC-46-0-s001.pdf]

Supporting Information for

Organodichalcogenide Structure and Stability: Hierarchical *Ab initio* Benchmark and DFT Performance Study

Steven E. Beutick,[a,b] Francesco Lambertini,[a] Trevor A. Hamlin,*[b] F. M. Bickelhaupt,* [b,c,d] Laura Orian*[a]

[a] S. E. Beutick, Francesco Lambertini, Prof. Dr. L. Orian
Dipartimento di Scienze Chimiche, Università degli Studi di Padova, Via Marzolo 1, 35129 Padova, Italy.
E-mail: laura.orian@unipd.it

[b] S. E. Beutick, Dr. T. A. Hamlin, Prof. Dr. F. M. Bickelhaupt
Department of Chemistry and Pharmaceutical Sciences, Amsterdam Institute of Molecular and Life Sciences (AIMMS), Vrije Universiteit Amsterdam, De Boelelaan 1108, 1081 HZ Amsterdam, The Netherlands.
E-mail: f.m.bickelhaupt@vu.nl, t.a.hamlin@vu.nl

[c] Prof. Dr. F. M. Bickelhaupt
Institute of Molecules and Materials, Radboud University, Heyendaalseweg 135, 6525 AJ Nijmegen, The Netherlands.

[d] Prof. Dr. F. M. Bickelhaupt
Department of Chemical Sciences, University of Johannesburg, Auckland Park, Johannesburg 2006, South Africa.

CONTENTS

Example input file

Table S1. Homolytic and heterolytic Ch^1–Ch^2 bond energies (in kcal mol^{-1}) for four model systems (CH_3S–$S(O)_nCH_3$ and CH_3Se–$Se(O)_nCH_3$ where n = 0 or 2). The bond dissociation energies were given for four systems (**SS**, **SSO2**, **SeSe**, **SeSeO2**) and for four ZORA-relativistic density functionals approximations that performed well in our benchmark (mPBE, mPW, MN15, M06) in combination with the Slater-type TZ2P basis set.

Table S2. Homolytic Ch^1–Ch^2 bond energies (in kcal mol^{-1}) with and without scalar relativistic effects considered by the ZORA approximation. The bond dissociation energies were given for four systems (**SS**, **SSO2**, **SeSe**, **SeSeO2**) and for four density functionals approximations that performed well in our benchmark (mPBE, mPW, MN15, M06) in combination with the Slater-type TZ2P basis set.

Figure S1. Energy of organodichalcogenide model systems CH_3Ch^1–$Ch^2(O)_nCH_3$ (Ch^1, Ch^2 = S, Se; n = 0, 1 2) as a function of the dihedral angle τ(C–Ch–Ch–C) computed at a) BP86, b) BP86-D3(BJ), and c) M06-2X.

Figure S2. The ZORA-CCSD(T)/BS2+ optimized geometries of the organodichalcogenide model systems. The bond distances (in Å), angles (in degrees), dihedral angle τ(C–Ch–Ch–C) (in degrees), and the pyramidalization of the methyl groups $\gamma(CH_xH_yH_z)$ are provided. The pyramidalization of the methyl groups $\gamma(CH_xH_yH_z)$ is defined as the deviation in the sum of the angle between the hydrogens from 180 degrees.

Figure S3. The ZORA-CCSD(T)/BS2+ optimized geometries (in Å, degrees) of the radical fragments that comprise the organodichalcogenide model systems. The pyramidalization of the methyl groups $\gamma(CH_xH_yH_z)$ is defined as the deviation in the sum of the angle between the hydrogen from 180 degrees.

Table S3. The deviation in percentage of the spin-squared operator computed at UCCSD(T)/BS#//UCCSD(T)/BS2+ for the MeCh$^•$ radicals relative to the spin-squared expectation value $\langle S^2 \rangle$ of a pure doublet state ((s(s + 1) for s = 1/2)).

Table S4. The deviation in the spin-squared operator computed at UCCSD(T)/BS#//UCCSD(T)/BS2+ for the MeCh$^•$ radicals relative to the spin-squared expectation value $\langle S^2 \rangle$ of a pure doublet state ((s(s + 1) for s = 1/2)).

Table S5. Homolytic Ch^1–Ch^2 bond energies ΔE (in kcal mol^{-1}) of organodichalcogenide model systems.

Table S6. Basis set superposition error BSSE (in kcal mol^{-1}) in the homolytic Ch^1–Ch^2 bond energies in kcal mol^{-1}) of organodichalcogenide model systems.

Table S7. The counterpoise corrected homolytic Ch^1–Ch^2 bond energies ΔE_{CPC} (in kcal mol^{-1}) of organodichalcogenide model systems.

Table S8. The convergence of the homolytic Ch^1–Ch^2 bond energy ΔE and counterpoise corrected bond energy ΔE_{CPC} (in kcal mol^{-1}) of organodichalcogenide model systems.

Table S9. Homolytic Ch^1–Ch^2 bond energies (ΔE, in kcal mol^{-1}) of organodichalcogenide model systems at various levels of theory.

Table S10. Homolytic Ch^1–Ch^2 bond energies (ΔE, in kcal mol^{-1}) of the organodichalcogenide model systems. All DFT values were computed at ZORA-[XC functional]/TZ2P//ZORA-[XC functional]/TZ2P.

Figure S4. The mean of the root-mean-square deviations (RMSD) for organodichalcogenide model systems and the fragments optimized at ZORA-[XC functional]/TZ2P//ZORA-[XC functional]/TZ2P compared to the ZORA-CCSD(T)/BS2+ optimized geometries. The XC functionals were ordered based on MAE in bond energy within their respective family of XC approximation.

Table S11. The logical statements considered in Table S12. The statements reflect the significant trends in the bond distance of organodichalcogenide model systems optimized at ZORA-CCSD(T)/BS2+.

Table S12. A truth table based (as denoted in Table S11) to verify the significant trends in the bond distance of organodichalcogenide model systems optimized at ZORA-[XC functional]/TZ2P// ZORA-[XC functional]/TZ2P as compared to the geometries optimized at ZORA-CCSD(T)/BS2+.

Table S13. The logical statements that were tested in Table S14. The statements reflect the trends in the counterpoise-corrected bond energies ΔE_{CPC} of the organodichalcogenide model systems at CCSD(T)/BS3+//CCSD(T)/BS2+.

Table S14. A truth table to verify the trend (as denoted in Table S13) in the homolytic Ch^1–Ch^2 bond energies of the organodichalcogenide model systems as compared to the trends in the counterpoise-corrected CCSD(T)/BS3+//CCSD(T)/BS2+ energies ΔE_{CPC}. All DFT values were computed at ZORA-[XC functional]/TZ2P//ZORA-[XC functional]/TZ2P.

Table S15. Error (in kcal mol^{-1}) in the bond energy ΔE of organodichalcogenide model systems at ZORA-[XC functional]/TZ2P//ZORA-[XC functional]/TZ2P relative to counterpoise-corrected ZORA-CCSD(T)/BS3+//ZORA-CCSD(T)/BS2+ energy ΔE_{CPC}.

Table S16. Absolute errors (in kcal mol^{-1}) in bond energy ΔE of organodichalcogenide model systems at ZORA-[XC functional]/TZ2P//ZORA-[XC functional]/TZ2P relative to counterpoise-corrected ZORA-CCSD(T)/BS3+//ZORA-CCSD(T)/BS2+ energy ΔE_{CPC}.

Table S17. Mean error ME, mean absolute error MAE and largest deviation LD (all in kcal mol^{-1}) and the species with the largest deviation in the homolytic Ch^1–Ch^2 bond energy out of organodichalcogenide model systems at ZORA-[XC functional]/TZ2P//ZORA-[XC functional]/TZ2P relative to ZORA-CCSD(T)/BS3+//ZORA-CCSD(T)/BS2+ energies ΔE_{CPC}.

Table S18. Mean error ME and mean absolute error MAE (both in kcal mol^{-1}) in homolytic Ch^1–Ch^2 bond energies of organodichalcogenides at ZORA-[XC functional]/TZ2P//ZORA-[XC functional]/TZ2P relative to the counterpoise-corrected ZORA-CCSD(T)/BS3+//ZORA-CCSD(T)/BS2+ energies ΔE_{CPC}, computed for the overall set of model systems and subcategories thereof.

Table S19 Cartesian coordinates (in Å) and total electronic energies (kcal mol^{-1}) of the organodichalcogenide model systems, computed at ZORA-CCSD(T)/BS3+//ZORA-CCSD(T)/BS2+ using ORCA 5.0. All species correspond to minima (no imaginary frequencies).

Table S20. Cartesian coordinates (in Å) and total electronic energies (kcal mol^{-1}) of radical fragments formed by homolytic dissociation of the organodichalcogenide model systems, computed at ZORA-CCSD(T)/BS3+//ZORA-CCSD(T)/BS2+ using ORCA 5.0. All species correspond to minima (no imaginary frequencies).

Example input file

```
Task GeometryOptimization

System
   Atoms
     [cartesian coordinates (Å)]
   End
End

GeometryOptimization
   MaxIterations 199
   Convergence
     Gradients 1e-05
     Energy 1e-05
   End
End

Properties
   NormalModes Yes
End
NormalModes
   Hessian Analytical
   ReScanFreqRange -1000 10.0
End

Engine ADF
   Basis
     Type [BASISSET]
     Core None
   End
   XC
     [FUNCTIONAL]
   End
   NumericalQuality VeryGood
   SCF
     Iterations 199
     Converge 1e-05
   End
EndEngine
```

Table S1. Homolytic and heterolytic Ch[1]–Ch[2] bond energies (in kcal mol^{-1}) for four model systems ($CH_3S–S(O)_nCH_3$ and $CH_3Se–Se(O)_nCH_3$ where n = 0 or 2). The bond dissociation energies were given for four systems (**SS, SSO2, SeSe, SeSeO2**) and for four ZORA-relativistic density functionals approximations that performed well in our benchmark (mPBE, mPW, MN15, M06) in combination with the Slater-type TZ2P basis set.

Complex	Fragment 1	Fragment 2	Δ*E* (ZORA-mPBE/TZ2P)	Δ*E* (ZORA-mPW/TZ2P)	Δ*E* (ZORA-MN15/TZ2P)	Δ*E* (ZORA-M06/TZ2P)
$CH_3S–SCH_3$	$CH_3S^•$	$^•SCH_3$	–64.6	–63.2	–66.1	–65.8
$CH_3S–SCH_3$	CH_3S^+	$^-SCH_3$	–256.1	–255.8	–256.2	–256.0
$CH_3S–S(O)_2CH_3$	$CH_3S^•$	$^•S(O)_2CH_3$	–51.7	–50.1	–60.0	–57.7
$CH_3S–S(O)_2CH_3$	CH_3S^+	$^-S(O)_2CH_3$	–232.8	–232.0	–235.6	–234.6
$CH_3S–S(O)_2CH_3$	CH_3S^-	$^+S(O)_2CH_3$	–213.7	–213.1	–221.8	–218.6
$CH_3Se–SeCH_3$	$CH_3Se^•$	$^•SeCH_3$	–58.3	–57.5	–53.8	–56.7
$CH_3Se–SeCH_3$	CH_3Se^+	$^-SeCH_3$	–246.9	–246.9	–247.1	–246.0
$CH_3Se–Se(O)_2CH_3$	$CH_3Se^•$	$^•Se(O)_2CH_3$	–37.1	–35.9	–40.9	–40.6
$CH_3Se–Se(O)_2CH_3$	CH_3Se^+	$^-Se(O)_2CH_3$	–204.3	–203.6	–205.1	–201.2
$CH_3Se–Se(O)_2CH_3$	CH_3Se^-	$^+Se(O)_2CH_3$	–210.9	–210.6	–219.9	–219.1

Table S2. Homolytic Ch[1]–Ch[2] bond energies (in kcal mol^{-1}) with and without scalar relativistic effects considered by the ZORA approximation. The bond dissociation energies were given for four systems (**SS**, **SSO2**, **SeSe**, **SeSeO2**) and for four density functionals approximations that performed well in our benchmark (mPBE, mPW, MN15, M06) in combination with the Slater-type TZ2P basis set.

	Level of Theory		
Dissociated bond	Δ*E* (ZORA-mPBE/TZ2P)	Δ*E* (mPBE /TZ2P)	$\Delta\Delta E_{ZORA}$
$CH_3S–SCH_3$	–64.6	–64.8	–0.2
$CH_3S–S(O)_2CH_3$	–51.7	–52.1	–0.4
$CH_3Se–SeCH_3$	–58.3	–58.0	0.3
$CH_3Se–Se(O)_2CH_3$	–37.1	–38.5	–1.3
	Δ*E* (ZORA-mPW/TZ2P)	Δ*E* (mPW /TZ2P)	$\Delta\Delta E_{ZORA}$
$CH_3S–SCH_3$	–63.2	–63.3	–0.2
$CH_3S–S(O)_2CH_3$	–50.1	–50.5	–0.4
$CH_3Se–SeCH_3$	–57.5	–57.2	0.3
$CH_3Se–Se(O)_2CH_3$	–35.9	–37.2	–1.3
	Δ*E* (ZORA-MN15/TZ2P)	Δ*E* (MN15/TZ2P)	$\Delta\Delta E_{ZORA}$
$CH_3S–SCH_3$	–66.1	–66.3	–0.2
$CH_3S–S(O)_2CH_3$	–60.0	–60.3	–0.3
$CH_3Se–SeCH_3$	–53.8	–53.4	0.4
$CH_3Se–Se(O)_2CH_3$	–40.9	–41.9	–1.0
	Δ*E* (ZORA-M06/TZ2P)	Δ*E* (M06/TZ2P)	$\Delta\Delta E_{ZORA}$
$CH_3S–SCH_3$	–65.8	–66.0	–0.2
$CH_3S–S(O)_2CH_3$	–57.7	–58.2	–0.5
$CH_3Se–SeCH_3$	–56.7	–56.5	0.2
$CH_3Se–Se(O)_2CH_3$	–40.6	–42.0	–1.3

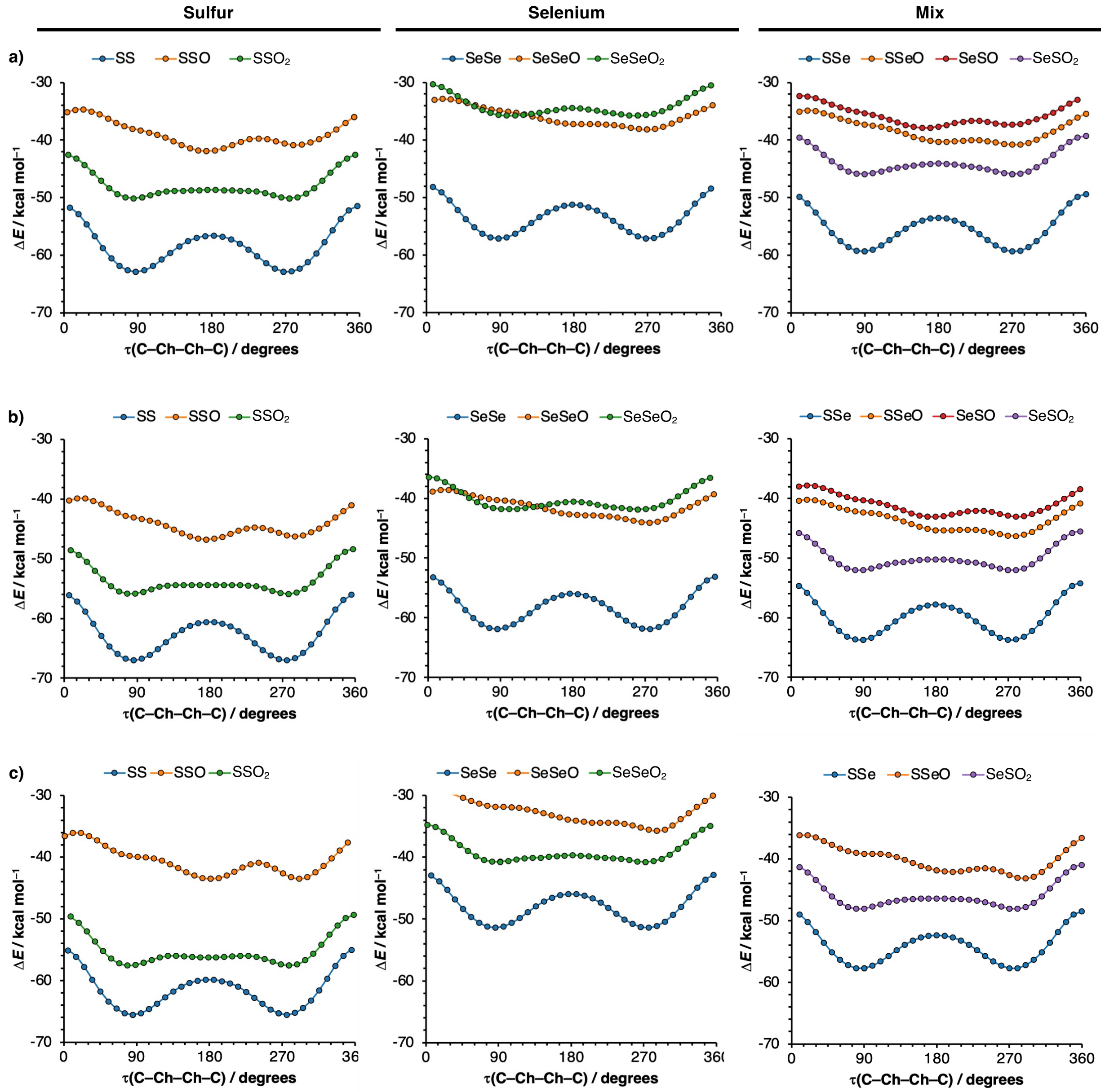

Figure S1. Energy of organodichalcogenide model systems CH_3Ch^1–$Ch^2(O)_nCH_3$ (Ch^1, Ch^2 = S, Se; n = 0, 1 2) as a function of the dihedral angle τ(C–Ch–Ch–C) computed at a) BP86, b) BP86-D3(BJ), and c) M06-2X.

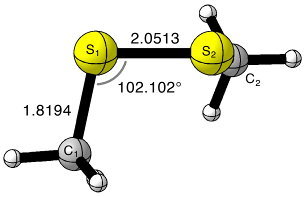

$r(S_2C_2)$ = 1.8194 Å
$\theta(S_1S_2C_2)$ = 102.102°
$\tau(C_1S_1S_2C_2)$ = 84.899°
$\gamma(C_1H_1H_2H_3)$ = 30.689°
$\gamma(C_1H_1H_2H_3)$ = 30.689°

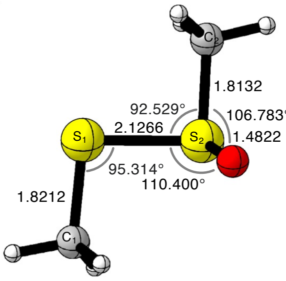

$\tau(C_1S_1S_2C_2)$ = 178.738°
$\tau(C_1S_1S_2O)$ = 72.310°
$\gamma(C_1H_1H_2H_3)$ = 30.689°
$\gamma(C_2H_4H_5H_6)$ = 29.773°

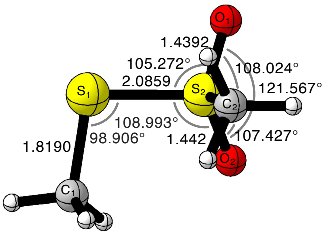

$r(S_2C_2)$ = 1.7800 Å
$\theta(S_1S_2C_2)$ = 104.287°
$\tau(C_1S_1S_2C_2)$ = 81.833°
$\tau(C_1S_1S_2O_1)$ = 164.556°
$\tau(C_1S_1S_2O_2)$ = 32.654°
$\gamma(C_1H_1H_2H_3)$ = 29.773°
$\gamma(C_2H_4H_5H_6)$ = 26.726°

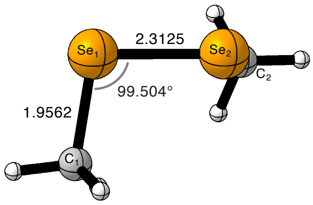

$r(Se_2C_2)$ = 1.9562 Å
$\theta(Se_1Se_2C_2)$ = 99.502°
$\tau(C_1Se_1Se_2C_2)$ = 85.049°
$\gamma(C_1H_1H_2H_3)$ = 28.981°
$\gamma(C_2H_4H_5H_6)$ = 28.982°

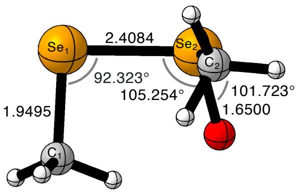

$r(Se_2C_2)$ = 1.9516 Å
$\theta(Se_1Se_2C_2)$ = 96.509°
$\tau(C_1Se_1Se_2C_2)$ = 76.989°
$\tau(C_1Se_1Se_2O)$ = 27.080°
$\gamma(C_1H_1H_2H_3)$ = 27.184°
$\gamma(C_2H_4H_5H_6)$ = 26.045°

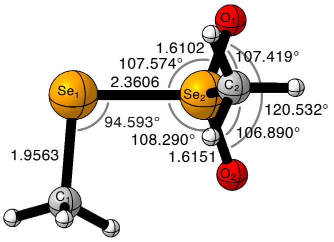

$r(Se_2C_2)$ = 1.9238 Å
$\theta(Se_1Se_2C_2)$ = 105.138°
$\tau(C_1Se_1Se_2C_2)$ = 87.308°
$\tau(C_1Se_1Se_2O_1)$ = 158.426°
$\tau(C_1Se_1Se_2O_2)$ = 26.672°
$\gamma(C_1H_1H_2H_3)$ = 27.278°
$\gamma(C_2H_4H_5H_6)$ = 23.760°

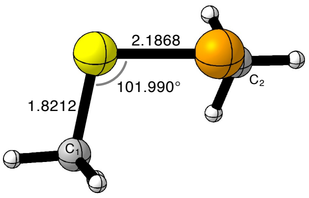

$r(SeC_2)$ = 1.9542 Å
$\theta(SSeC_2)$ = 99.422°
$\tau(C_1SSeC_2)$ = 84.954°
$\gamma(C_1H_1H_2H_3)$ = 30.850°
$\gamma(C_2H_4H_5H_6)$ = 28.937°

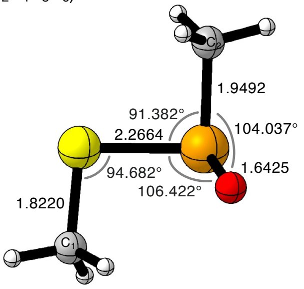

$\tau(C_1SSeC_2)$ = 163.020°
$\tau(C_1SSeO)$ = 57.947°
$\gamma(C_1H_1H_2H_3)$ = 30.081°
$\gamma(C_2H_4H_5H_6)$ = 25.723°

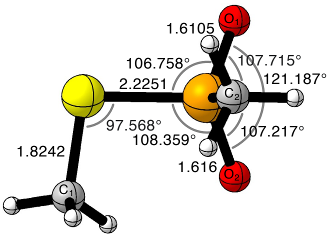

$r(SeC_2)$ = 1.9219 Å
$\theta(SSeC_2)$ = 104.408°
$\tau(C_1SSeC_2)$ = 87.114°
$\tau(C_1SSeO_1)$ = 158.966°
$\tau(C_1SSeO_2)$ = 26.919°
$\gamma(C_1H_1H_2H_3)$ = 29.234°
$\gamma(C_2H_4H_5H_6)$ = 23.825°

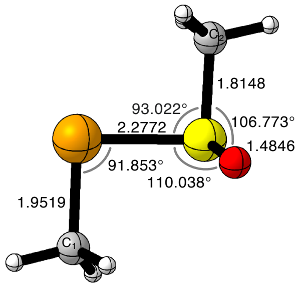

$\tau(C_1SeSC_2)$ = 177.380°
$\tau(C_1SeSO)$ = 68.302°
$\gamma(C_1H_1H_2H_3)$ = 28.510°
$\gamma(C_2H_4H_5H_6)$ = 27.550°

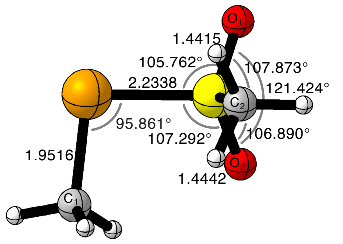

$r(SC_2)$ = 1.7821 Å
$\theta(SeSC_2)$ = 104.530°
$\tau(C_1SeSC_2)$ = 83.215°
$\tau(C_1SeSO_1)$ = 163.045°
$\tau(C_1SeSO_2)$ = 31.134°
$\gamma(C_1H_1H_2H_3)$ = 28.010°
$\gamma(C_2H_4H_5H_6)$ = 26.494°

Figure S2. The ZORA-CCSD(T)/BS2+ optimized geometries of the organodichalcogenide model systems. The bond distances (in Å), angles (in degrees), dihedral angle τ(C–Ch–Ch–C) (in degrees), and the pyramidalization of the methyl groups γ(CHxHyHz) are provided. The pyramidalization of the methyl groups γ(CHxHyHz) is defined as the deviation in the sum of the angle between the hydrogens from 180 degrees.

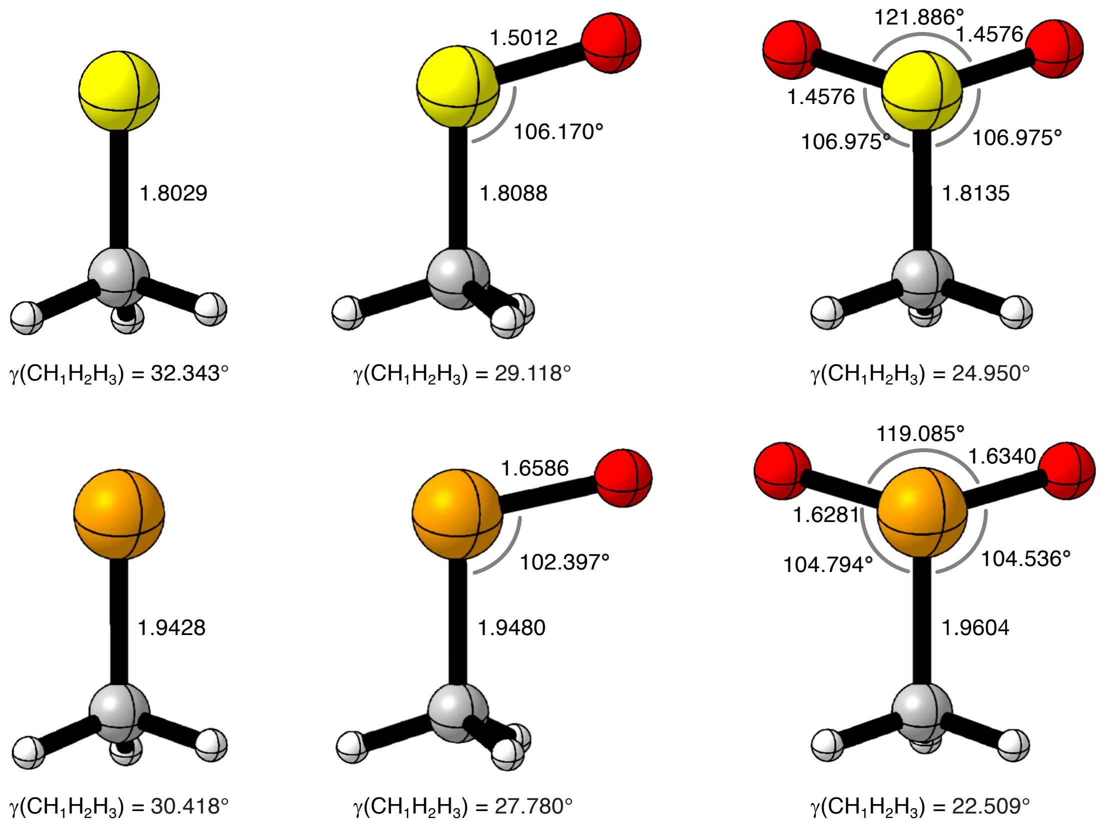

Figure S3. The ZORA-CCSD(T)/BS2+ optimized geometries (in Å, degrees) of the radical fragments that comprise the organodichalcogenide model systems. The pyramidalization of the methyl groups $\gamma(CH_xH_yH_z)$ is defined as the deviation in the sum of the angle between the hydrogen from 180 degrees.

Table S3. The deviation in percentage of the spin-squared operator computed at UCCSD(T)/BS#//UCCSD(T)/BS2+ for the MeCh• radicals relative to the spin-squared expectation value ⟨S^2⟩ of a pure doublet state ((s(s + 1) for s = 1/2)).

Basis set	MeS•	MeOS•	Me(O)$_2$S•	MeSe•	MeOSe•	Me(O)$_2$Se•
ZORA-def2-SVP	1.6%	3.5%	4.8%	1.5%	3.5%	7.0%
ZORA-def2-TZVPP	2.5%	6.2%	5.6%	2.0%	5.6%	8.6%
ZORA-def2-QZVPP	2.7%	6.7%	5.7%	2.1%	5.9%	8.6%
ma-ZORA-def2-SVP	1.6%	4.5%	5.0%	1.6%	5.0%	7.9%
ma-ZORA-def2-TZVPP	2.5%	6.4%	5.6%	2.0%	6.0%	8.7%
ma-ZORA-def2-QZVPP	2.7%	6.7%	5.7%	2.1%	6.0%	8.7%

Table S4. The deviation in the spin-squared operator computed at UCCSD(T)/BS#//UCCSD(T)/BS2+ for the MeCh• radicals relative to the spin-squared expectation value ⟨S^2⟩ of a pure doublet state ((s(s + 1) for s = 1/2)).

Basis set	MeS•	MeOS•	Me(O)$_2$S•	MeSe•	MeOSe•	Me(O)$_2$Se•
ZORA-def2-SVP	0.0118	0.0261	0.0359	0.0116	0.0264	0.0527
ZORA-def2-TZVPP	0.0190	0.0466	0.0417	0.0152	0.0420	0.0644
ZORA-def2-QZVPP	0.0202	0.0500	0.0425	0.0160	0.0443	0.0646
ma-ZORA-def2-SVP	0.0123	0.0341	0.0377	0.0119	0.0373	0.0596
ma-ZORA-def2-TZVPP	0.0191	0.0483	0.0418	0.0153	0.0448	0.0651
ma-ZORA-def2-QZVPP	0.0202	0.0504	0.0426	0.0160	0.0453	0.0649

Table S5. Homolytic Ch1–Ch2 bond energies Δ*E* (in kcal mol^{-1}) of organodichalcogenide model systems.[a]

Method	Basis set	SS	SSO	SSO_2	SeSe	SeSeO	$SeSeO_2$	SSe	SSeO	$SSeO_2$	SeSO	$SeSO_2$
HF	BS1	–29.4	–4.8	–28.1	–24.6	–0.6	–15.1	–26.3	–3.3	–18.7	0.6	–22.1
	BS2	–32.7	–12.4	–33.8	–25.3	–3.1	–17.1	–28.2	–7.6	–21.5	–5.9	–26.3
	BS3	–32.6	–12.5	–33.5	–25.3	–3.1	–16.8	–28.1	–7.8	–21.1	–5.9	–26.1
MP2	BS1	–59.7	–39.4	–51.3	–53.4	–43.1	–55.1	–55.8	–45.0	–59.3	–34.6	–45.3
	BS2	–69.6	–52.5	–64.3	–61.1	–52.3	–63.3	–64.8	–55.9	–67.8	–46.4	–57.5
	BS3	–72.5	–55.8	–66.9	–63.9	–54.8	–65.2	–67.5	–58.6	–69.5	–49.6	–60.1
CCSD	BS1	–53.4	–28.3	–44.8	–47.1	–27.1	–33.8	–49.6	–29.1	–38.0	–23.4	–38.8
	BS2	–59.5	–38.7	–54.8	–52.4	–32.9	–40.1	–55.3	–36.0	–44.2	–33.1	–48.4
	BS3	–61.9	–41.3	–57.1	–53.9	–34.5	–41.5	–57.2	–38.2	–45.7	–35.2	–50.3
CCSD(T)	BS1	–56.3	–32.0	–46.6	–50.1	–31.7	–35.8	–52.6	–33.3	–39.8	–27.4	–40.8
	BS2	–63.5	–43.2	–57.4	–56.2	–38.4	–42.7	–59.3	–41.2	–46.8	–37.8	–51.2
	BS3	–66.2	–46.2	–59.9	–58.4	–40.4	–44.4	–61.7	–43.7	–48.5	–40.4	–53.4
HF	BS1+	–28.2	–6.1	–27.3	–24.0	–2.0	–15.8	–25.6	–5.3	–19.1	–1.1	–22.0
	BS2+	–32.5	–12.4	–33.3	–25.2	–2.9	–16.7	–28.0	–7.6	–21.0	–5.9	–25.9
	BS3+	–32.6	–12.5	–33.4	–25.2	–3.1	–16.7	–28.0	–7.8	–21.0	–5.9	–26.0
MP2	BS1+	–59.4	–41.3	–51.5	–53.5	–44.4	–56.0	–55.9	–47.5	–60.2	–36.5	–45.9
	BS2+	–69.7	–52.8	–63.8	–61.2	–52.3	–63.2	–64.8	–56.3	–67.7	–46.7	–57.2
	BS3+	–72.5	–55.8	–66.8	–63.9	–54.9	–65.2	–67.5	–58.7	–69.4	–49.7	–60.0
CCSD	BS1+	–52.7	–29.9	–44.8	–46.9	–27.7	–34.4	–49.4	–30.7	–38.5	–25.1	–39.3
	BS2+	–59.5	–38.8	–54.4	–52.4	–32.7	–39.9	–55.3	–36.2	–43.8	–33.2	–48.1
	BS3+	–61.9	–41.3	–57.0	–54.0	–34.5	–41.4	–57.2	–38.2	–45.6	–35.3	–50.3
CCSD(T)	BS1+	–55.7	–33.7	–46.8	–50.1	–32.4	–36.6	–52.5	–35.1	–40.6	–29.2	–41.5
	BS2+	–63.5	–43.4	–56.9	–56.3	–38.3	–42.5	–59.3	–41.5	–46.5	–38.0	–50.9
	BS3+	–66.2	–46.2	–59.8	–58.5	–40.4	–44.3	–61.7	–43.8	–48.4	–40.5	–53.4

[a] Computed at ZORA-Method/BS#//ZORA-CCSD(T)/BS2+.

Table S6. Basis set superposition error BSSE (in kcal mol^{-1}) in the homolytic Ch1–Ch2 bond energies in kcal mol^{-1}) of organodichalcogenide model systems.[a]

Method	Basis set	SS	SSO	SSO_2	SeSe	SeSeO	$SeSeO_2$	SSe	SSeO	$SSeO_2$	SeSO	$SeSO_2$
HF	BS1	2.9	4.1	5.6	1.5	2.6	3.5	2.2	3.3	4.2	3.3	4.9
	BS2	0.5	0.6	0.9	0.6	0.8	0.9	0.6	0.6	0.8	0.7	0.9
	BS3	0.1	0.1	0.2	0.1	0.2	0.2	0.1	0.1	0.2	0.1	0.2
MP2	BS1	7.3	8.2	10.2	4.5	6.6	7.1	5.9	7.5	8.6	6.8	8.7
	BS2	2.0	2.3	3.0	3.2	3.7	3.9	2.7	3.0	3.4	3.0	3.7
	BS3	0.9	1.0	1.3	1.9	2.1	2.1	1.4	1.6	1.7	1.6	1.9
CCSD	BS1	8.0	8.6	10.6	4.9	6.7	7.1	6.5	7.9	8.8	7.0	8.9
	BS2	1.9	2.2	2.8	2.9	3.4	3.6	2.5	2.7	3.1	2.7	3.4
	BS3	0.6	0.8	1.0	1.5	1.7	1.8	1.1	1.2	1.4	1.3	1.5
CCSD(T)	BS1	8.4	9.0	11.1	5.1	7.2	7.6	6.7	8.2	9.0	7.5	9.4
	BS2	2.1	2.4	3.1	3.1	3.7	3.9	2.7	3.0	3.5	3.0	3.7
	BS3	0.7	0.9	1.2	1.6	1.9	2.0	1.3	1.4	1.6	1.4	1.7
HF	BS1+	1.7	2.6	3.6	1.0	1.6	2.1	1.4	2.0	2.5	2.2	3.3
	BS2+	0.3	0.3	0.4	0.4	0.4	0.5	0.4	0.3	0.4	0.4	0.5
	BS3+	0.1	0.1	0.1	0.1	0.1	0.1	0.1	0.1	0.1	0.1	0.1
MP2	BS1+	6.4	7.1	8.7	4.1	5.4	5.8	5.3	6.3	7.8	5.8	7.3
	BS2+	1.8	2.1	2.4	3.1	3.3	3.7	2.5	2.7	3.2	2.7	3.2
	BS3+	0.8	1.0	1.2	1.9	2.0	2.0	1.4	1.5	1.6	1.5	1.8
CCSD	BS1+	6.9	7.4	9.0	4.4	5.5	6.0	5.7	6.5	7.6	6.0	7.5
	BS2+	1.7	1.9	2.3	2.8	3.0	3.2	2.4	2.4	2.7	2.5	2.9
	BS3+	0.6	0.7	0.9	1.5	1.6	1.7	1.1	1.2	1.3	1.2	1.5
CCSD(T)	BS1+	7.5	7.9	9.7	4.8	6.0	6.5	6.2	7.1	8.2	6.5	8.1
	BS2+	2.0	2.2	2.6	3.1	3.3	3.5	2.6	2.7	3.1	2.7	3.2
	BS3+	0.7	0.8	1.0	1.7	1.8	1.9	1.3	1.3	1.4	1.4	1.6

[a] Computed at ZORA-Method/BS#//ZORA-CCSD(T)/BS2+.

Table S7. The counterpoise corrected homolytic Ch1–Ch2 bond energies Δ*ECPC* (in kcal mol^{-1}) of organodichalcogenide model systems.[a]

Method	Basis set	SS	SSO	SSO_2	SeSe	SeSeO	$SeSeO_2$	SSe	SSeO	$SSeO_2$	SeSO	$SeSO_2$
HF	BS1	–26.5	–0.7	–22.5	–23.1	2.0	–11.6	–24.1	0.0	–14.6	3.9	–17.2
	BS2	–32.2	–11.8	–32.9	–24.7	–2.3	–16.2	–27.6	–7.0	–20.6	–5.2	–25.4
	BS3	–32.5	–12.4	–33.3	–25.2	–3.0	–16.6	–28.0	–7.7	–20.9	–5.8	–25.9
MP2	BS1	–52.3	–31.1	–41.1	–49.0	–36.5	–48.0	–49.9	–37.4	–50.7	–27.7	–36.6
	BS2	–67.7	–50.2	–61.3	–58.0	–48.6	–59.4	–62.1	–53.0	–64.4	–43.5	–53.8
	BS3	–71.7	–54.7	–65.6	–62.0	–52.8	–63.1	–66.0	–57.0	–67.7	–48.0	–58.2
CCSD	BS1	–45.4	–19.8	–34.2	–42.3	–20.4	–26.7	–43.1	–21.2	–29.2	–16.4	–29.9
	BS2	–57.7	–36.5	–52.0	–49.4	–29.5	–36.5	–52.9	–33.3	–41.0	–30.3	–45.1
	BS3	–61.3	–40.5	–56.1	–52.4	–32.8	–39.7	–56.1	–36.9	–44.3	–33.9	–48.8
CCSD(T)	BS1	–47.9	–23.0	–35.5	–45.0	–24.6	–28.2	–45.8	–25.2	–30.8	–20.0	–31.5
	BS2	–61.4	–40.7	–54.3	–53.1	–34.7	–38.8	–56.6	–38.2	–43.3	–34.8	–47.5
	BS3	–65.5	–45.3	–58.8	–56.8	–38.6	–42.4	–60.4	–42.3	–46.9	–39.0	–51.7
HF	BS1+	–26.5	–3.5	–23.7	–23.0	–0.3	–13.8	–24.2	–3.2	–16.5	1.1	–18.7
	BS2+	–32.2	–12.1	–32.8	–24.7	–2.5	–16.2	–27.6	–7.3	–20.5	–5.5	–25.4
	BS3+	–32.5	–12.5	–33.3	–25.2	–3.0	–16.6	–28.0	–7.8	–20.9	–5.9	–25.9
MP2	BS1+	–52.9	–34.2	–42.8	–49.4	–39.1	–50.2	–50.6	–41.2	–52.3	–30.6	–38.6
	BS2+	–67.8	–50.7	–61.4	–58.1	–49.0	–59.6	–62.3	–53.6	–64.5	–44.0	–54.0
	BS3+	–71.7	–54.8	–65.6	–62.0	–52.9	–63.2	–66.0	–57.2	–67.7	–48.1	–58.3
CCSD	BS1+	–45.8	–22.5	–35.8	–42.5	–22.3	–28.5	–43.6	–24.2	–30.9	–19.1	–31.8
	BS2+	–57.8	–36.9	–52.1	–49.5	–29.7	–36.7	–53.0	–33.7	–41.1	–30.8	–45.2
	BS3+	–61.3	–40.6	–56.1	–52.4	–32.9	–39.7	–56.1	–37.0	–44.3	–34.0	–48.8
CCSD(T)	BS1+	–48.2	–25.7	–37.1	–45.2	–26.4	–30.1	–46.2	–28.0	–32.4	–22.7	–33.4
	BS2+	–61.5	–41.2	–54.4	–53.2	–35.0	–39.0	–56.7	–38.7	–43.4	–35.3	–47.6
	BS3+	–65.5	–45.4	–58.8	–56.8	–38.7	–42.5	–60.4	–42.5	–46.9	–39.1	–51.8

[a] Computed at ZORA-Method/BS#//ZORA-CCSD(T)/BS2+.

Table S8. The convergence of the homolytic Ch^1–Ch^2 bond energy ΔE and counterpoise corrected bond energy ΔE_{CPC} (in kcal mol^{-1}) of organodichalcogenide model systems.[a]

	**Basis set convergence**		**Method convergence**	
Energy	ΔE	ΔE_{cpc}	ΔE	ΔE_{cpc}
Comparison 1	CCSD(T)/**BS2+**	CCSD(T)/**BS2+**	**CCSD**/BS3+	**CCSD**/BS3+
Comparison 2	CCSD(T)/**BS3+**	CCSD(T)/**BS3+**	**CCSD(T)**/BS3+	**CCSD(T)**/BS3+
**SS**	–2.7	–4.0	–4.3	–4.2
**SSO**	–2.8	–4.2	–4.9	–4.8
**SSO$_2$**	–2.9	–4.4	–2.8	–2.7
**SeSe**	–2.2	–3.6	–4.5	–4.4
**SeSeO**	–2.1	–3.7	–5.9	–5.8
**SeSeO$_2$**	–1.8	–3.5	–2.9	–2.8
**SSe**	–2.4	–3.7	–4.5	–4.3
**SSeO**	–2.3	–3.8	–5.6	–5.5
**SSeO$_2$**	–1.9	–3.5	–2.8	–2.6
**SeSO**	–2.5	–3.8	–5.2	–5.1
**SeSO$_2$**	–2.5	–4.2	–3.1	–3.0
Min	–1.8	–3.5	–2.8	–2.6
Max	–2.9	–4.4	–5.9	–5.8
Mean	–2.4	–3.9	–4.2	–4.1

Trend in DFT bond energies at the respective DFT equilibrium geometries

Table S9. Homolytic Ch1–Ch2 bond energies (Δ*E*, in kcal mol^{-1}) of organodichalcogenide model systems at various levels of theory.

	BP86			BP86-D3(BJ)			M06-2X		
	**TZ2P[a]**	**QZ4P[b]**	**ΔΔ*E*[c]**	**TZ2P[d]**	**QZ4P[e]**	**ΔΔ*E*[f]**	**TZ2P[g]**	**QZ4P[h]**	**ΔΔ*E*[i]**
**SS**	–62.8	–63.7	0.9	–67.0	–67.9	–0.9	–65.6	–66.6	–1.0
**SSO**	–41.9	–42.9	1.0	–46.8	–47.8	–1.0	–43.5	–44.8	–1.3
**SSO2**	–50.1	–51.0	0.9	–55.9	–56.8	–1.0	–57.6	–58.6	–1.0
**SeSe**	–57.1	–56.2	–0.9	–61.8	–60.9	0.9	–51.4	–50.1	1.3
**SeSeO**	–38.2	–37.2	–1.0	–44.1	–43.0	1.1	–35.7	–34.2	1.5
**SeSeO2**	–35.8	–34.8	–1.0	–41.8	–40.8	1.0	–40.8	–39.2	1.6
**Sse**	–59.3	–59.2	–0.1	–63.7	–63.7	0.1	–57.8	–57.4	0.3
**SSeO**	–39.3	–40.0	0.7	–45.4	–45.1	0.3	–42.1	–41.7	0.4
**SSeO2**	–36.6	–37.7	1.1	–43.6	–43.4	0.2	–48.3	–47.9	0.4
**SeSO**	–37.9	–38.0	0.1	–43.1	–43.2	–0.1	–34.5	–34.3	0.1
**SeSO2**	–45.9	–45.7	–0.2	–52.0	–51.8	0.2	–48.1	–47.5	0.6

[a] Computed at BP86/TZ2P//BP86/TZ2P
[b] Computed at BP86/QZ4P// BP86/QZ4P
[c] Difference in bond energy: ΔΔ*E* = ΔE(BP86/QZ4P//BP86/QZ4P) – ΔE(BP86/TZ2P//BP86/TZ2P)
[d] Computed at BP86-D3(BJ)/TZ2P//BP86-D3(BJ)/TZ2P
[e] Computed at BP86-D3(BJ)/QZ4P//BP86-D3(BJ)/QZ4P
[f] Difference in bond energy: ΔΔ*E* = ΔE(BP86-D3(BJ)/QZ4P//BP86-D3(BJ)/QZ4P) – ΔE(BP86-D3(BJ)/TZ2P// BP86-D3(BJ)/TZ2P)
[g] Computed at M06-2X/TZ2P//M06-2X/TZ2P
[h] Computed at M06-2X/QZ4P//M06-2X/QZ4P
[i] Difference in bond energy: ΔΔ*E* = ΔE(M06-2X/QZ4P//M06-2X/QZ4P) – ΔE(M06-2X/TZ2P// M06-2X/TZ2P)

Table S10. Homolytic Ch[1]–Ch[2] bond energies (ΔE, in kcal mol^{-1}) of the organodichalcogenide model systems. All DFT values were computed at ZORA-[XC functional]/TZ2P//ZORA-[XC functional]/TZ2P.

Method/ Functional	SS	SSO	SSO2	SeSe	SeSeO	SeSeO2	SSe	SSeO	SSeO2	SeSO	SeSO2
CCSD(T)/BS3+// CCSD(T)/BS2+	–66.2	–46.2	–59.8	–58.5	–40.4	–44.3	–61.7	–43.8	–48.4	–40.5	–53.4
VWN	–81.5	–60.8	–70.9	–73.6	–56.6	–54.0	–76.8	–60.1	–57.1	–55.6	–65.4
BLYP	–58.3	–37.1	–44.3	–53.6	–34.2	–30.7	–55.3	–36.0	–32.2	–33.8	–40.9
OLYP	–60.0	–38.1	–46.2	–51.3	–31.5	–30.6	–54.9	–36.2	–34.3	–32.5	–40.6
BP86	–62.9	–41.9	–50.1	–57.1	–38.2	–35.8	–59.3	–40.3	–37.9	–37.9	–45.9
PBE	–66.1	–45.1	–53.5	–59.9	–41.0	–38.8	–62.3	–43.3	–41.2	–40.7	–48.9
OPBE	–64.9	–43.0	–51.8	–55.1	–35.1	–35.7	–59.3	–40.7	–40.1	–36.7	–45.3
RPBE	–60.5	–39.2	–47.0	–54.1	–34.8	–32.8	–56.6	–37.9	–35.3	–34.9	–42.5
revPBE	–60.9	–39.6	–47.4	–54.6	–35.2	–33.2	–57.1	–38.2	–35.6	–35.3	–42.9
HTBS	–65.7	–44.2	–52.4	–60.2	–40.1	–38.1	–62.2	–42.5	–40.2	–40.3	–48.3
PW91	–66.3	–45.3	–53.7	–60.3	–41.4	–39.0	–62.6	–43.6	–41.3	–41.1	–49.3
mPBE	–64.6	–43.5	–51.7	–58.3	–39.3	–37.1	–60.7	–41.9	–39.6	–39.1	–47.2
mPW	–63.2	–42.0	–50.1	–57.5	–38.3	–35.9	–59.6	–40.5	–38.0	–38.0	–46.0
BLYP-D3(BJ)	–63.2	–42.9	–51.2	–58.8	–40.8	–37.4	–60.4	–41.9	–38.7	–39.8	–48.0
BP86-D3(BJ)	–67.0	–46.8	–55.9	–61.8	–44.0	–41.8	–63.7	–45.4	–43.6	–43.1	–52.0
PBE-D3(BJ)	–68.4	–47.8	–56.8	–62.2	–44.1	–41.9	–64.6	–46.1	–44.2	–43.5	–52.3
M06-L	–65.3	–43.7	–55.5	–56.0	–37.5	–37.5	–60.3	–41.6	–42.3	–37.2	–48.7
SCAN	–63.4	–43.8	–55.5	–58.6	–40.3	–40.4	–60.4	–42.2	–42.5	–39.8	–51.1
r2SCAN	–63.7	–43.8	–54.9	–57.5	–39.4	–39.4	–59.8	–41.6	–42.0	–39.3	–50.1
SCAN-D3(BJ)	–64.0	–44.5	–56.4	–59.2	–41.1	–41.2	–60.9	–42.9	–43.3	–40.5	–52.0
B3LYP	–57.9	–36.2	–47.3	–52.2	–32.2	–32.9	–54.3	–34.5	–35.2	–32.0	–42.8
PBE0	–63.5	–42.2	–54.8	–56.6	–37.1	–39.8	–59.3	–40.0	–43.0	–36.9	–49.2
OPBE0	–62.7	–40.9	–53.5	–53.3	–33.2	–37.7	–57.2	–38.4	–42.5	–34.3	–46.5
MPW1PW	–61.1	–39.7	–52.1	–54.7	–34.9	–37.4	–57.1	–37.6	–40.3	–34.7	–46.8
MN15	–66.1	–45.0	–60.0	–53.8	–36.8	–40.9	–59.1	–41.8	–47.0	–37.0	–51.4
M06	–65.8	–45.4	–57.7	–56.7	–38.8	–40.6	–61.0	–43.4	–45.7	–38.9	–50.9
M06-2X	–65.6	–43.5	–57.6	–51.4	–35.7	–40.8	–57.8	–42.1	–48.3	–34.5	–48.1
M06-HF	–67.8	–44.1	–58.7	–54.2	–38.1	–45.0	–59.8	–43.2	–51.8	–35.6	–49.7
CAM-B3LYP	–58.6	–36.8	–50.4	–51.6	–31.6	–35.0	–54.4	–34.5	–38.3	–31.4	–44.8
M11	–62.6	–40.9	–54.0	–57.0	–37.4	–40.7	–58.9	–38.5	–43.0	–36.6	–49.3
MN12-SX	–66.0	–44.6	–58.6	–58.4	–37.7	–41.0	–61.4	–40.1	–44.3	–39.4	–52.8
wB97X	–62.7	–40.6	–55.4	–54.5	–34.4	–39.2	–57.9	–38.1	–43.5	–34.3	–48.8
LCY-PBE	–66.4	–45.4	–62.2	–57.5	–39.0	–46.1	–61.2	–42.7	–50.8	–38.6	–55.0
CCSD(T)/BS2+	–63.4	–43.3	–56.8	–55.9	–38.0	–42.1	–59.1	–41.3	–46.2	–37.8	–50.6

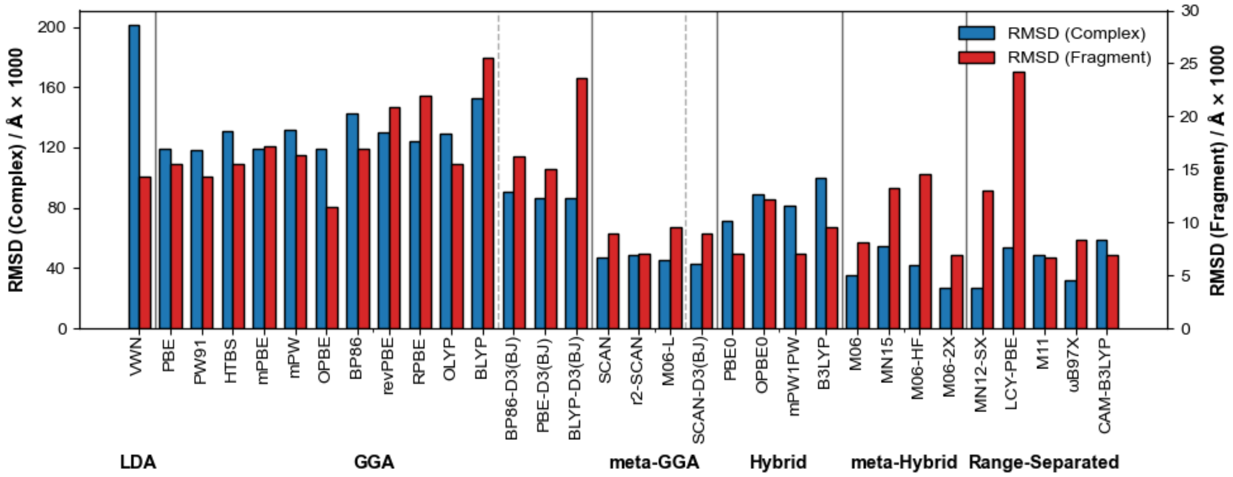

Figure S4. The mean of the root-mean-square deviations (RMSD) for organodichalcogenide model systems and the fragments optimized at ZORA-[XC functional]/TZ2P//ZORA-[XC functional]/TZ2P compared to the ZORA-CCSD(T)/BS2+ optimized geometries. The XC functionals were ordered based on MAE in bond energy within their respective family of XC approximation.

Trend in DFT-equilibrium geometries

For the DFT benchmark, we compared the trends in the Ch–Ch bond distance found in the CCSD(T)/BS2+ optimized geometries of the organodichalcogenide model systems, including the oxidation of equal Ch–Ch (Ch–Ch to ChChO to $ChChO_2$) bond and the different bonds within the same oxidation state, by means of logical statements. For example, the trend in bond distance for the oxidation of the SS ($SS(O)_n$ for n = 0, 1, 2) goes from r(**SS**) = 2.05 to r(**SSO**) = 2.13 to r(**SSO$_2$**) = 2.09 Å. The trend in the bond distance, relative to each other, can be described by logical statements, namely r(**SS**) < r(**SSO$_2$**) and r(**SSO$_2$**) < r(**SSO**). The following statements were included (Table S11):

Table S11. The logical statements considered in Table S12. The statements reflect the significant trends in the bond distance of organodichalcogenide model systems optimized at ZORA-CCSD(T)/BS2+.

**Trends**	**Statement 1:**	**Statement 2:**	**Statement 3:**
Oxidation (SS)	r(**SS**) < r(**SSO$_2$**)	r(**SSO$_2$**) < r(**SSO**)	
Oxidation (SeSe)	r(**SeSe**) < r(**SeSeO$_2$**)	r(**SeSeO$_2$**) < r(**SeSeO**)	
Oxidation (SSe)	r(**SSe**) < r(**SSeO$_2$**)	r(**SSeO$_2$**) < r(**SSeO**)	
Oxidation (SeS)	r(**SeS**) < r(**SeSO$_2$**)	r(**SeSO$_2$**) < r(**SeSO**)	
Internal (ChCh)	r(**SS**) < r(**SSe**)	r(**SSe**) < r(**SeSe**)	
Internal (ChChO)	r(**SSO**) < r(**SSeO**)	r(**SSeO**) < r(**SeSeO**)	r(**SSeO**) < r(**SeSO**)
Internal ($ChChO_2$)	r(**SSO$_2$**) < r(**SeSO$_2$**)	r(**SSeO$_2$**) < r(**SeSeO$_2$**)	r(**SSeO$_2$**) < r(**SeSO$_2$**)

Table S12. A truth table based (as denoted in Table S11) to verify the significant trends in the bond distance of organodichalcogenide model systems optimized at ZORA-[XC functional]/TZ2P// ZORA-[XC functional]/TZ2P as compared to the geometries optimized at ZORA-CCSD(T)/BS2+.

	Oxidation ($SS(O)_n$)	Oxidation ($SeSe(O)_n$)	Oxidation ($SSe(O)_n$)	Oxidation ($SeS(O)_n$)	Internal (ChCh)	Internal (ChChO)	Internal ($ChChO_2$)
CCSD(T)/BS2+	TRUE	TRUE	TRUE	TRUE	TRUE	TRUE	TRUE
VWN	TRUE	TRUE	TRUE	TRUE	TRUE	FALSE	FALSE
BP86	TRUE	TRUE	TRUE	TRUE	TRUE	TRUE	FALSE
BLYP	TRUE	TRUE	TRUE	TRUE	TRUE	TRUE	FALSE
PW91	TRUE	TRUE	TRUE	TRUE	TRUE	TRUE	FALSE
PBE	TRUE	TRUE	TRUE	TRUE	TRUE	TRUE	FALSE
RPBE	TRUE	TRUE	TRUE	TRUE	TRUE	TRUE	FALSE
revPBE	TRUE	TRUE	TRUE	TRUE	TRUE	TRUE	FALSE
mPBE	TRUE	TRUE	TRUE	TRUE	TRUE	TRUE	FALSE
mPW	TRUE	TRUE	TRUE	TRUE	TRUE	TRUE	FALSE
HTBS	TRUE	TRUE	TRUE	TRUE	TRUE	TRUE	FALSE
OLYP	TRUE	TRUE	TRUE	TRUE	TRUE	TRUE	FALSE
OPBE	TRUE	TRUE	TRUE	TRUE	TRUE	TRUE	FALSE
BLYP-D3(BJ)	TRUE	TRUE	TRUE	TRUE	TRUE	TRUE	FALSE
BP86-D3(BJ)	TRUE	TRUE	TRUE	TRUE	TRUE	TRUE	FALSE
PBE-D3(BJ)	TRUE	TRUE	TRUE	TRUE	TRUE	TRUE	FALSE
M06-L	TRUE	TRUE	TRUE	TRUE	TRUE	TRUE	TRUE
SCAN	TRUE	TRUE	TRUE	TRUE	TRUE	TRUE	FALSE
SCAN-D3(BJ)	TRUE	TRUE	TRUE	TRUE	TRUE	TRUE	FALSE
r2-SCAN	TRUE	TRUE	TRUE	TRUE	TRUE	TRUE	FALSE
B3LYP	TRUE	TRUE	TRUE	TRUE	TRUE	TRUE	TRUE
mPW1PW	TRUE	TRUE	TRUE	TRUE	TRUE	TRUE	TRUE
PBE0	TRUE	TRUE	TRUE	TRUE	TRUE	TRUE	TRUE
OPBE0	TRUE	TRUE	TRUE	TRUE	TRUE	TRUE	TRUE
M06	TRUE	TRUE	TRUE	TRUE	TRUE	TRUE	TRUE
M06-2X	TRUE	TRUE	TRUE	TRUE	TRUE	TRUE	TRUE
M06-HF	TRUE	TRUE	TRUE	TRUE	TRUE	TRUE	TRUE
MN15	TRUE	TRUE	TRUE	TRUE	TRUE	TRUE	TRUE
CAM-B3LYP	TRUE	TRUE	TRUE	TRUE	TRUE	TRUE	TRUE
M11	TRUE	TRUE	TRUE	TRUE	TRUE	TRUE	TRUE
MN12-SX	TRUE	TRUE	TRUE	TRUE	TRUE	TRUE	TRUE
wB97X	TRUE	TRUE	TRUE	TRUE	TRUE	TRUE	TRUE
LCY-PBE	TRUE	TRUE	TRUE	TRUE	TRUE	TRUE	TRUE

Trend in DFT bond energies at the respective DFT equilibrium geometries

For the DFT benchmark, we compared the trends that were found in the CCSD(T)/BS3+//CCSD(T)/BS2+, including the oxidation of equal Ch–Ch (Ch–Ch to ChChO to $ChChO_2$) bond and the different bonds within the same oxidation state, by means of logical statements. For example, the trend in the homolytic bond energies for the oxidation of the SS ($SS(O)_n$ for n = 0, 1, 2) goes from ΔE (**SS**) = –66.2 to ΔE (**SSO**) = –46.2 to ΔE (**SSO_2**) = –59.8. The trend in the energy, relative to each other, can be described by logical statements, namely ΔE (**SS**) < ΔE (**SSO_2**) and ΔE (**SSO_2**) < ΔE (**SSO**). For Internal ChChO, the comparison between SeSeO and SeSO was excluded because the energy difference is within 0.1 kcal mol^{-1}. These included the following (Table S13):

Table S13. The logical statements that were tested in Table S14. The statements reflect the trends in the counterpoise-corrected bond energies ΔE_{CPC} of the organodichalcogenide model systems at CCSD(T)/BS3+//CCSD(T)/BS2+.

Trends	Statement 1:	Statement 2:	Statement 3:
Oxidation (SS)	ΔE (**SS**) < ΔE (**SSO_2**)	ΔE (**SSO_2**) < ΔE (**SSO**)	
Oxidation (SeSe)	ΔE (**SeSe**) < ΔE (**$SeSeO_2$**)	ΔE (**$SeSeO_2$**) < ΔE (**SeSeO**)	
Oxidation (SSe)	ΔE (**SSe**) < ΔE (**$SSeO_2$**)	ΔE (**$SSeO_2$**) < ΔE (**SSeO**)	
Oxidation (SeS)	ΔE (**SeS**) < ΔE (**$SeSO_2$**)	ΔE (**$SeSO_2$**) < ΔE (**SeSO**)	
Internal (ChCh)	ΔE (**SS**) < ΔE (**SSe**)	ΔE (**SSe**) < ΔE (**SeSe**)	
Internal (ChChO)	ΔE (**SSO**) < ΔE (**SSeO**)	ΔE (**SSeO**) < ΔE (**SeSeO**)	ΔE (**SSeO**) < ΔE (**SeSO**)
Internal ($ChChO_2$)	ΔE (**SSO_2**) < ΔE (**$SeSO_2$**)	ΔE (**SSeO**) < ΔE (**SeSeO**)	ΔE (**SSeO**) < ΔE (**SeSO**)

Table S14. A truth table to verify the trend (as denoted in Table S13) in the homolytic Ch^1–Ch^2 bond energies of the organodichalcogenide model systems as compared to the trends in the counterpoise-corrected CCSD(T)/BS3+//CCSD(T)/BS2+ energies ΔE_{CPC}. All DFT values were computed at ZORA-[XC functional]/TZ2P//ZORA-[XC functional]/TZ2P.

	**Oxidation ($SS(O)_n$)**	**Oxidation ($SeSe(O)_n$)**	**Oxidation ($SSe(O)_n$)**	**Oxidation ($SeS(O)_n$)**	**Internal (ChCh)**	**Internal (ChChO)**	**Internal ($ChChO_2$)**
**CCSD(T)/BS3+// CCSD(T)/BS2+**	TRUE	TRUE	TRUE	TRUE	TRUE	TRUE	TRUE
VWN	TRUE	FALSE	FALSE	TRUE	TRUE	TRUE	TRUE
BLYP	TRUE	FALSE	FALSE	TRUE	TRUE	TRUE	TRUE
OLYP	TRUE	FALSE	FALSE	TRUE	TRUE	TRUE	TRUE
BP86	TRUE	FALSE	FALSE	TRUE	TRUE	TRUE	TRUE
PBE	TRUE	FALSE	FALSE	TRUE	TRUE	TRUE	TRUE
OPBE	TRUE	TRUE	FALSE	TRUE	TRUE	TRUE	TRUE
RPBE	TRUE	FALSE	FALSE	TRUE	TRUE	TRUE	TRUE
revPBE	TRUE	FALSE	FALSE	TRUE	TRUE	TRUE	TRUE
HTBS	TRUE	FALSE	FALSE	TRUE	TRUE	TRUE	TRUE
PW91	TRUE	FALSE	FALSE	TRUE	TRUE	TRUE	TRUE
mPBE	TRUE	FALSE	FALSE	TRUE	TRUE	TRUE	TRUE
mPW	TRUE	FALSE	FALSE	TRUE	TRUE	TRUE	TRUE
BLYP-D3(BJ)	TRUE	FALSE	FALSE	TRUE	TRUE	TRUE	TRUE
BP86-D3(BJ)	TRUE	FALSE	FALSE	TRUE	TRUE	TRUE	TRUE
PBE-D3(BJ)	TRUE	FALSE	FALSE	TRUE	TRUE	TRUE	TRUE
M06-L	TRUE	TRUE	TRUE	TRUE	TRUE	TRUE	TRUE
SCAN	TRUE	TRUE	TRUE	TRUE	TRUE	TRUE	TRUE
r2SCAN	TRUE	TRUE	TRUE	TRUE	TRUE	TRUE	TRUE
SCAN-D3(BJ)	TRUE	TRUE	TRUE	TRUE	TRUE	TRUE	TRUE
B3LYP	TRUE	TRUE	TRUE	TRUE	TRUE	TRUE	TRUE
PBE0	TRUE	TRUE	TRUE	TRUE	TRUE	TRUE	TRUE
OPBE0	TRUE	TRUE	TRUE	TRUE	TRUE	TRUE	TRUE
MPW1PW	TRUE	TRUE	TRUE	TRUE	TRUE	TRUE	TRUE
MN15	TRUE	TRUE	TRUE	TRUE	TRUE	TRUE	TRUE
M06	TRUE	TRUE	TRUE	TRUE	TRUE	TRUE	TRUE
M06-2X	TRUE	TRUE	TRUE	TRUE	TRUE	TRUE	FALSE
M06-HF	TRUE	TRUE	TRUE	TRUE	TRUE	TRUE	FALSE
CAM-B3LYP	TRUE	TRUE	TRUE	TRUE	TRUE	TRUE	TRUE
M11	TRUE	TRUE	TRUE	TRUE	TRUE	TRUE	TRUE
MN12-SX	TRUE	TRUE	TRUE	TRUE	TRUE	TRUE	TRUE
wB97X	TRUE	TRUE	TRUE	TRUE	TRUE	TRUE	TRUE
LCY-PBE	TRUE	TRUE	TRUE	TRUE	TRUE	TRUE	TRUE
CCSD(T)/BS2+	TRUE	TRUE	TRUE	TRUE	TRUE	TRUE	TRUE

Table S15. Error (in kcal mol^{-1}) in the bond energy ΔE of organodichalcogenide model systems at ZORA-[XC functional]/TZ2P//ZORA-[XC functional]/TZ2P relative to counterpoise-corrected ZORA-CCSD(T)/BS3+//ZORA-CCSD(T)/BS2+ energy ΔE_{CPC}.

Method/ Functional	SS	SSO	SSO2	SeSe	SeSeO	SeSeO2	SSe	SSeO	SSeO2	SeSO	SeSO2
CCSD(T)/BS3+// CCSD(T)/BS2+	0.0	0.0	0.0	0.0	0.0	0.0	0.0	0.0	0.0	0.0	0.0
VWN	–16.0	–15.5	–12.1	–16.8	–17.9	–11.5	–16.4	–17.6	–10.1	–16.5	–13.6
BLYP	7.2	8.3	14.5	3.3	4.5	11.8	5.2	6.5	14.7	5.3	10.9
OLYP	5.5	7.3	12.6	5.6	7.2	11.8	5.5	6.3	12.6	6.5	11.2
BP86	2.7	3.5	8.7	–0.3	0.5	6.7	1.1	2.2	9.0	1.2	5.9
PBE	–0.6	0.3	5.3	–3.0	–2.3	3.7	–1.9	–0.9	5.8	–1.6	2.8
OPBE	0.7	2.4	7.0	1.7	3.5	6.8	1.2	1.8	6.8	2.4	6.5
RPBE	5.1	6.1	11.8	2.7	3.9	9.7	3.8	4.6	11.6	4.2	9.3
revPBE	4.7	5.8	11.4	2.2	3.5	9.3	3.4	4.3	11.3	3.8	8.8
HTBS	–0.2	1.1	6.4	–3.3	–1.4	4.4	–1.8	0.0	6.7	–1.2	3.5
PW91	–0.7	0.1	5.1	–3.5	–2.8	3.4	–2.2	–1.1	5.7	–2.0	2.4
mPBE	0.9	1.9	7.1	–1.5	–0.6	5.4	–0.3	0.6	7.4	0.0	4.6
mPW	2.4	3.3	8.7	–0.7	0.4	6.6	0.8	1.9	9.0	1.0	5.8
BLYP-D3(BJ)	2.3	2.4	7.6	–1.9	–2.1	5.1	0.1	0.6	8.3	–0.7	3.8
BP86-D3(BJ)	–1.5	–1.4	2.9	–5.0	–5.4	0.7	–3.3	–2.9	3.4	–4.0	–0.3
PBE-D3(BJ)	–2.9	–2.4	2.0	–5.4	–5.4	0.6	–4.2	–3.6	2.7	–4.4	–0.5
M06-L	0.3	1.7	3.3	0.8	1.2	5.0	0.2	0.9	4.7	1.9	3.1
SCAN	2.2	1.5	3.3	–1.8	–1.6	2.1	0.1	0.3	4.4	–0.7	0.6
r2SCAN	1.9	1.6	3.9	–0.7	–0.7	3.0	0.6	0.8	4.9	–0.2	1.7
SCAN-D3(BJ)	1.6	0.8	2.4	–2.4	–2.4	1.3	–0.5	–0.4	3.6	–1.4	–0.2
B3LYP	7.6	9.1	11.5	4.6	6.4	9.6	6.1	8.0	11.7	7.1	8.9
PBE0	2.0	3.2	4.0	0.3	1.5	2.7	1.2	2.5	4.0	2.1	2.6
OPBE0	2.8	4.4	5.3	3.6	5.4	4.7	3.2	4.0	4.4	4.8	5.2
MPW1PW	4.4	5.7	6.7	2.2	3.8	5.1	3.3	4.8	6.6	4.4	5.0
MN15	–0.6	0.4	–1.2	3.0	1.9	1.6	1.3	0.7	–0.1	2.1	0.3
M06	–0.3	–0.1	1.1	0.1	–0.2	1.9	–0.5	–0.9	1.2	0.2	0.8
M06-2X	0.0	1.9	1.2	5.4	2.9	1.7	2.7	0.4	–1.4	4.6	3.6
M06-HF	–2.3	1.2	0.1	2.6	0.5	–2.5	0.7	–0.8	–4.9	3.5	2.0
CAM-B3LYP	6.9	8.6	8.4	5.2	7.0	7.4	6.1	8.0	8.7	7.7	6.9
M11	3.0	4.4	4.8	–0.2	1.3	1.7	1.5	4.0	3.9	2.5	2.4
MN12-SX	–0.5	0.7	0.2	–1.6	1.0	1.5	–0.9	2.3	2.7	–0.3	–1.1
wB97X	2.8	4.8	3.4	2.3	4.3	3.2	2.5	4.3	3.5	4.8	2.9
LCY-PBE	–0.9	–0.1	–3.4	–0.7	–0.3	–3.6	–0.7	–0.2	–3.9	0.5	–3.2
CCSD(T)/BS2+	4.0	4.1	4.4	3.6	3.7	3.4	3.7	3.7	3.5	3.8	4.1

Table S16. Absolute errors (in kcal mol^{-1}) in bond energy ΔE of organodichalcogenide model systems at ZORA-[XC functional]/TZ2P//ZORA-[XC functional]/TZ2P relative to counterpoise-corrected ZORA-CCSD(T)/BS3+//ZORA-CCSD(T)/BS2+ energy ΔE_{CPC}.

Method/ Functional	SS	SSO	SSO2	SeSe	SeSeO	SeSeO2	SSe	SSeO	SSeO2	SeSO	SeSO2
CCSD(T)/BS3+// CCSD(T)/BS2+	0.0	0.0	0.0	0.0	0.0	0.0	0.0	0.0	0.0	0.0	0.0
VWN	16.0	15.5	12.1	16.8	17.9	11.5	16.4	17.6	10.1	16.5	13.6
BLYP	7.2	8.3	14.5	3.3	4.5	11.8	5.2	6.5	14.7	5.3	10.9
OLYP	5.5	7.3	12.6	5.6	7.2	11.8	5.5	6.3	12.6	6.5	11.2
BP86	2.7	3.5	8.7	0.3	0.5	6.7	1.1	2.2	9.0	1.2	5.9
PBE	0.6	0.3	5.3	3.0	2.3	3.7	1.9	0.9	5.8	1.6	2.8
OPBE	0.7	2.4	7.0	1.7	3.5	6.8	1.2	1.8	6.8	2.4	6.5
RPBE	5.1	6.1	11.8	2.7	3.9	9.7	3.8	4.6	11.6	4.2	9.3
revPBE	4.7	5.8	11.4	2.2	3.5	9.3	3.4	4.3	11.3	3.8	8.8
HTBS	0.2	1.1	6.4	3.3	1.4	4.4	1.8	0.0	6.7	1.2	3.5
PW91	0.7	0.1	5.1	3.5	2.8	3.4	2.2	1.1	5.7	2.0	2.4
mPBE	0.9	1.9	7.1	1.5	0.6	5.4	0.3	0.6	7.4	0.0	4.6
mPW	2.4	3.3	8.7	0.7	0.4	6.6	0.8	1.9	9.0	1.0	5.8
BLYP-D3(BJ)	2.3	2.4	7.6	1.9	2.1	5.1	0.1	0.6	8.3	0.7	3.8
BP86-D3(BJ)	1.5	1.4	2.9	5.0	5.4	0.7	3.3	2.9	3.4	4.0	0.3
PBE-D3(BJ)	2.9	2.4	2.0	5.4	5.4	0.6	4.2	3.6	2.7	4.4	0.5
M06-L	0.3	1.7	3.3	0.8	1.2	5.0	0.2	0.9	4.7	1.9	3.1
SCAN	2.2	1.5	3.3	1.8	1.6	2.1	0.1	0.3	4.4	0.7	0.6
r2SCAN	1.9	1.6	3.9	0.7	0.7	3.0	0.6	0.8	4.9	0.2	1.7
SCAN-D3(BJ)	1.6	0.8	2.4	2.4	2.4	1.3	0.5	0.4	3.6	1.4	0.2
B3LYP	7.6	9.1	11.5	4.6	6.4	9.6	6.1	8.0	11.7	7.1	8.9
PBE0	2.0	3.2	4.0	0.3	1.5	2.7	1.2	2.5	4.0	2.1	2.6
OPBE0	2.8	4.4	5.3	3.6	5.4	4.7	3.2	4.0	4.4	4.8	5.2
MPW1PW	4.4	5.7	6.7	2.2	3.8	5.1	3.3	4.8	6.6	4.4	5.0
MN15	0.6	0.4	1.2	3.0	1.9	1.6	1.3	0.7	0.1	2.1	0.3
M06	0.3	0.1	1.1	0.1	0.2	1.9	0.5	0.9	1.2	0.2	0.8
M06-2X	0.0	1.9	1.2	5.4	2.9	1.7	2.7	0.4	1.4	4.6	3.6
M06-HF	2.3	1.2	0.1	2.6	0.5	2.5	0.7	0.8	4.9	3.5	2.0
CAM-B3LYP	6.9	8.6	8.4	5.2	7.0	7.4	6.1	8.0	8.7	7.7	6.9
M11	3.0	4.4	4.8	0.2	1.3	1.7	1.5	4.0	3.9	2.5	2.4
MN12-SX	0.5	0.7	0.2	1.6	1.0	1.5	0.9	2.3	2.7	0.3	1.1
wB97X	2.8	4.8	3.4	2.3	4.3	3.2	2.5	4.3	3.5	4.8	2.9
LCY-PBE	0.9	0.1	3.4	0.7	0.3	3.6	0.7	0.2	3.9	0.5	3.2
CCSD(T)/BS2+	4.0	4.1	4.4	3.6	3.7	3.4	3.7	3.7	3.5	3.8	4.1

Table S17. Mean error ME, mean absolute error MAE and largest deviation LD (all in kcal mol^{-1}) and the species with the largest deviation in the homolytic Ch^1–Ch^2 bond energy out of organodichalcogenide model systems at ZORA-[XC functional]/TZ2P//ZORA-[XC functional]/TZ2P relative to ZORA-CCSD(T)/BS3+//ZORA-CCSD(T)/BS2+ energies ΔE_{CPC}.

Method	Functional	ME	MAE	LD	LD species
LDA	VWN	–14.92	14.92	17.92	SSeO2
GGA	PBE	0.69	2.56	5.75	SSeO2
GGA	PW91	0.40	2.63	5.66	SSeO2
GGA	HTBS	1.29	2.72	6.70	SSeO2
GGA	mPBE	2.30	2.74	7.35	SSeO2
GGA	mPW	3.56	3.69	8.96	SSeO2
GGA	OPBE	3.70	3.70	7.03	SeSeO2
GGA	BP86	3.75	3.80	9.04	SSeO2
GGA	revPBE	6.22	6.22	11.42	SSeO2
GGA	RPBE	6.62	6.62	11.84	SSeO2
GGA	OLYP	8.38	8.38	12.62	SSeO2
GGA	BLYP	8.38	8.38	14.75	SSeO2
GGA	BP86-D3(BJ)	–1.53	2.80	5.38	SSeO2
GGA	PBE-D3(BJ)	–2.14	3.10	5.40	SSeO2
GGA	BLYP-D3(BJ)	2.30	3.17	8.26	SSeO2
meta-GGA	SCAN	0.93	1.69	4.43	SSeO2
meta-GGA	r2SCAN	1.52	1.82	4.92	SSeO2
meta-GGA	M06-L	2.09	2.09	4.97	SeSeO2
meta-GGA	SCAN-D3(BJ)	0.22	1.55	3.62	SSeO2
Hybrid	PBE0	2.37	2.37	4.02	SSeO2
Hybrid	OPBE0	4.36	4.36	5.42	SeSeO
Hybrid	MPW1PW	4.72	4.72	6.73	SSeO2
meta-Hybrid	M06	0.31	0.67	1.87	SeSeO2
meta-Hybrid	MN15	0.86	1.20	3.03	SeSe
meta-Hybrid	M06-HF	0.02	1.92	4.85	SeSO
meta-Hybrid	M06-2X	2.10	2.35	5.41	SeSe
Range-Separated	MN12-SX	0.36	1.16	2.67	SeSO
Range-Separated	LCY-PBE	–1.51	1.60	3.92	SSeO2
Range-Separated	M11	2.67	2.71	4.80	SSO2
Range-Separated	ωB97X	3.55	3.55	4.81	SeSO
Range-Separated	CAM-B3LYP	7.35	7.35	8.67	SSeO2

Table S18. Mean error ME and mean absolute error MAE (both in kcal mol^{-1}) in homolytic Ch^1–Ch^2 bond energies of organodichalcogenides at ZORA-[XC functional]/TZ2P//ZORA-[XC functional]/TZ2P relative to the counterpoise-corrected ZORA-CCSD(T)/BS3+//ZORA-CCSD(T)/BS2+ energies ΔE_{CPC}, computed for the overall set of model systems and subcategories thereof.

Method/ Functional	Total Set		Ox = +1		Ox = +3		Ox = +5		Sulfur		Selenium		Mix	
	ME	MAE	ME	MAE	ME	MAE	ME	MAE	ME	MAE	ME	MAE	ME	MAE
CCSD(T)/BS3+// CCSD(T)/BS2+	0.0	0.0	0.0	0.0	0.0	0.0	0.0	0.0	0.0	0.0	0.0	0.0	0.0	0.0
VWN	–14.9	14.9	–16.4	16.4	–16.9	16.9	–11.8	11.8	–14.5	14.5	–15.4	15.4	–14.9	14.9
BLYP	8.4	8.4	5.2	5.2	6.1	6.1	13.0	13.0	10.0	10.0	6.5	6.5	8.5	8.5
OLYP	8.4	8.4	5.5	5.5	6.8	6.8	12.1	12.1	8.5	8.5	8.2	8.2	8.4	8.4
BP86	3.7	3.8	1.2	1.4	1.8	1.8	7.6	7.6	5.0	5.0	2.3	2.5	3.9	3.9
PBE	0.7	2.6	–1.8	1.8	–1.1	1.3	4.4	4.4	1.7	2.1	–0.5	3.0	0.8	2.6
OPBE	3.7	3.7	1.2	1.2	2.5	2.5	6.8	6.8	3.4	3.4	4.0	4.0	3.7	3.7
RPBE	6.6	6.6	3.9	3.9	4.7	4.7	10.6	10.6	7.7	7.7	5.4	5.4	6.7	6.7
revPBE	6.2	6.2	3.4	3.4	4.3	4.3	10.2	10.2	7.3	7.3	5.0	5.0	6.3	6.3
HTBS	1.3	2.7	–1.8	1.8	–0.4	0.9	5.2	5.2	2.4	2.5	–0.1	3.0	1.4	2.6
PW91	0.4	2.6	–2.1	2.1	–1.5	1.5	4.2	4.2	1.5	2.0	–0.9	3.2	0.6	2.7
mPBE	2.3	2.7	–0.3	0.9	0.4	0.8	6.1	6.1	3.3	3.3	1.1	2.5	2.4	2.6
mPW	3.6	3.7	0.8	1.3	1.7	1.7	7.5	7.5	4.8	4.8	2.1	2.6	3.7	3.7
BLYP-D3(BJ)	2.3	3.2	0.2	1.4	0.0	1.5	6.2	6.2	4.1	4.1	0.3	3.0	2.4	2.7
BP86-D3(BJ)	–1.5	2.8	–3.3	3.3	–3.4	3.4	1.7	1.8	0.0	1.9	–3.2	3.7	–1.4	2.8
PBE-D3(BJ)	–2.1	3.1	–4.2	4.2	–4.0	4.0	1.2	1.5	–1.1	2.5	–3.4	3.8	–2.0	3.1
M06-L	2.1	2.1	0.4	0.4	1.4	1.4	4.0	4.0	1.8	1.8	2.3	2.3	2.2	2.2
SCAN	0.9	1.7	0.1	1.3	–0.1	1.0	2.6	2.6	2.3	2.3	–0.5	1.8	0.9	1.2
r2SCAN	1.5	1.8	0.6	1.1	0.4	0.8	3.4	3.4	2.4	2.4	0.6	1.5	1.6	1.7
SCAN-D3(BJ)	0.2	1.5	–0.4	1.5	–0.8	1.3	1.8	1.9	1.6	1.6	–1.2	2.0	0.2	1.2
B3LYP	8.2	8.2	6.1	6.1	7.7	7.7	10.4	10.4	9.4	9.4	6.9	6.9	8.4	8.4
PBE0	2.4	2.4	1.1	1.1	2.3	2.3	3.3	3.3	3.1	3.1	1.5	1.5	2.5	2.5
OPBE0	4.4	4.4	3.2	3.2	4.7	4.7	4.9	4.9	4.2	4.2	4.6	4.6	4.3	4.3
MPW1PW	4.7	4.7	3.3	3.3	4.7	4.7	5.8	5.8	5.6	5.6	3.7	3.7	4.8	4.8
MN15	0.9	1.2	1.3	1.7	1.3	1.3	0.2	0.8	–0.5	0.7	2.2	2.2	0.9	0.9
M06	0.3	0.7	–0.2	0.3	–0.2	0.3	1.2	1.2	0.2	0.5	0.6	0.7	0.2	0.7
M06-2X	2.1	2.4	2.7	2.7	2.5	2.5	1.3	2.0	1.0	1.0	3.4	3.4	2.0	2.5
M06-HF	0.0	1.9	0.3	1.9	1.1	1.5	–1.3	2.4	–0.3	1.2	0.2	1.9	0.1	2.4
CAM-B3LYP	7.3	7.3	6.1	6.1	7.8	7.8	7.8	7.8	8.0	8.0	6.6	6.6	7.5	7.5
M11	2.7	2.7	1.4	1.6	3.1	3.1	3.2	3.2	4.1	4.1	0.9	1.1	2.9	2.9
MN12-SX	0.4	1.2	–1.0	1.0	0.9	1.1	0.8	1.3	0.2	0.5	0.3	1.3	0.5	1.5
wB97X	3.5	3.5	2.6	2.6	4.6	4.6	3.3	3.3	3.7	3.7	3.3	3.3	3.6	3.6
LCY-PBE	–1.5	1.6	–0.8	0.8	0.0	0.3	–3.6	3.6	–1.5	1.5	–1.6	1.6	–1.5	1.7
CCSD(T)/BS2+	3.8	3.8	3.8	3.8	3.8	3.8	3.9	3.9	4.2	4.2	3.6	3.6	3.8	3.8

Table S19 Cartesian coordinates (in Å) and total electronic energies (kcal mol^{-1}) of the organodichalcogenide model systems, computed at ZORA-CCSD(T)/BS3+//ZORA-CCSD(T)/BS2+ using ORCA 5.0. All species correspond to minima (no imaginary frequencies).

$CH_3S–SCH_3$
E = –551423.2592

S	-0.00634358	0.00782022	0.00678683
C	-0.38835145	-1.77050977	-0.03766225
H	-0.02145556	-2.22273588	-0.95775645
H	-1.47624060	-1.85201840	-0.00613223
H	0.04250827	-2.26988099	0.82767651
S	2.04498435	0.00714726	0.00754426
C	2.42704192	-0.10697973	-1.76766602
H	3.51493257	-0.07863631	-1.85031868
H	1.99617148	0.73810075	-2.30059379
H	2.06018659	-1.04410915	-2.18347919

$CH_3S–S(O)CH_3$
E = –598621.5151

S	-0.00598180	0.00690424	0.04055728
C	-0.18334918	-1.80099615	0.13314339
H	-1.25024899	-2.00230797	0.21951881
H	0.32824406	-2.17674183	1.01757810
H	0.20393022	-2.27433726	-0.76760870
S	2.12051601	-0.00450636	0.02208706
O	2.64688939	-0.36235339	1.36063926
C	2.20908886	1.80178825	-0.10958502
H	1.76197169	2.11963699	-1.05107202
H	3.26965774	2.04954209	-0.08330457
H	1.69788102	2.24067241	0.74634541

$CH_3S–S(O)_2CH_3$
E = –645842.5635

S	0.00003280	0.00582283	-0.00131831
C	-0.28569703	-1.78993942	0.04758700
H	-1.35532553	-1.92115584	-0.11406852
H	-0.01353419	-2.20894509	1.01478758
H	0.27426674	-2.26819485	-0.75168749
S	2.08590733	0.00093455	-0.00465857
O	2.46745231	1.32785106	-0.41090636
O	2.55138577	-1.16768424	-0.70995971
C	2.52741353	-0.19901262	1.70797547
H	2.12704666	-1.14470065	2.06802584
H	2.13051660	0.64430269	2.26747060
H	3.61723300	-0.21107741	1.73595549

$CH_3Se–SeCH_3$
E = –3118353.3478

Se	-0.00052113	0.00602241	0.00997161
C	-0.31930151	-1.92327719	-0.04515570

H	-1.40105086	-2.05637544	-0.01704094
H	0.13981270	-2.39054872	0.82161868
H	0.08069452	-2.33892985	-0.96660976
Se	2.31201055	0.01121552	0.00579072
C	2.63170123	-0.09900335	-1.92100106
H	2.23183466	-1.03193343	-2.31034807
H	3.71351049	-0.07475481	-2.05437111
H	2.17284336	0.75408486	-2.41305536

$CH_3Se–Se(O)CH_3$
E = –3165538.1303

Se	-0.03568400	0.00829400	0.06263800
C	-0.13121400	-1.92888900	0.25906600
H	-0.88873200	-2.28232500	-0.44105500
H	-0.39019000	-2.19736600	1.28333500
H	0.85700500	-2.31668800	-0.02144600
Se	2.37168400	-0.01904700	-0.00492600
O	2.77251700	-1.50629200	-0.59656500
C	2.64398200	-0.26809600	1.91149900
H	2.23724200	0.59267800	2.44744700
H	3.72220700	-0.36335800	2.06099600
H	2.13257600	-1.19119600	2.18834400

$CH_3Se–Se(O)_2CH_3$
E = –3212719.8694

Se	-0.00101300	0.00178500	-0.00179700
C	-0.15549800	-1.93636800	0.21484600
H	0.67203900	-2.39571600	-0.32099100
H	-1.10541000	-2.20917100	-0.24046800
H	-0.14526700	-2.20377900	1.26753300
Se	2.35959200	0.00371300	-0.00838300
O	2.84266000	1.36026000	-0.72899200
O	2.86613700	-1.43404300	-0.54196200
C	2.86704300	0.12310300	1.84344800
H	2.45935300	-0.74814700	2.35029400
H	2.46211400	1.05541500	2.22855800
H	3.95552000	0.11949600	1.84670300

$CH_3S–SeCH_3$
E = –1834887.6422

S	-0.00391686	0.01045294	0.00552776
C	-0.38290667	-1.77031612	-0.04088528
H	-0.00934737	-2.22205710	-0.95843071
H	-1.47099305	-1.85293730	-0.01663483
H	0.04148613	-2.27198573	0.82621998
Se	2.18290794	0.00972552	0.00259389
C	2.50019054	-0.10931673	-1.92199198
H	3.58158210	-0.08193034	-2.05792550
H	2.03689050	0.74067575	-2.41541293
H	2.10337973	-1.04555188	-2.30682939

$CH_3S–Se(O)CH_3$

E = –1882072.5292

S	-0.00865872	0.00994159	0.03087600
C	-0.17260490	-1.79436897	0.22387911
H	-1.21470789	-1.98272703	0.47753334
H	0.46554553	-2.12449695	1.04314596
H	0.07803050	-2.31583670	-0.69845033
Se	2.25768032	-0.00845047	0.03756513
O	2.71222754	-0.70127064	1.45569898
C	2.31914014	1.90485371	0.40502449
H	1.78204415	2.43991700	-0.37719159
H	3.37086293	2.18454998	0.42130225
H	1.86059739	2.04411847	1.38128064

$CH_3S–Se(O)_2CH_3$

E = –1882072.5292

S	0.04160763	0.03404393	-0.06694111
C	-0.22541563	-1.76771896	0.03381386
H	-1.24382248	-1.93113384	-0.31659250
H	-0.12643226	-2.12562940	1.05649645
H	0.48140508	-2.27001205	-0.62307025
Se	2.26598793	0.00377452	-0.01890433
O	2.76307345	1.40161298	-0.64563384
O	2.76880973	-1.40881388	-0.62069341
C	2.70409930	0.01318163	1.85239695
H	2.26719332	-0.87841378	2.29572456
H	2.29598129	0.92914426	2.27152056
H	3.79121363	-0.00693641	1.89798106

$CH_3Se–S(O)CH_3$

E = –1882084.6848

Se	0.00471620	0.00428991	0.05434643
C	-0.06647231	-1.93839049	0.22942915
H	-1.10797649	-2.19346934	0.41445411
H	0.54434879	-2.23185193	1.08019010
H	0.27789632	-2.41062668	-0.68707300
S	2.28173837	-0.00775029	0.02583170
O	2.80499453	-0.40832096	1.35613862
C	2.38598283	1.80225678	-0.05461722
H	1.92183885	2.14904476	-0.97809063
H	3.44884101	2.04239021	-0.04594813
H	1.89740890	2.22376004	0.82279886

$CH_3Se–S(O)_2CH_3$

E = –1929305.0203

Se	0.02857400	0.02104800	-0.04189200
C	-0.19475600	-1.91682800	0.01776300
H	-1.23435200	-2.10372000	-0.24578700
H	0.01131600	-2.29685200	1.01502900
H	0.47063900	-2.35760200	-0.71900400
S	2.26201000	-0.00574600	-0.01285200
O	2.67508400	1.30273900	-0.45463800
O	2.72069900	-1.20390600	-0.67591200

C	2.68492700	-0.15961900	1.71148900
H	2.27108200	-1.09157600	2.09135000
H	2.28793100	0.70197300	2.24246500
H	3.77464500	-0.17841000	1.75039000

Table S20. Cartesian coordinates (in Å) and total electronic energies (kcal mol^{-1}) of radical fragments formed by homolytic dissociation of the organodichalcogenide model systems, computed at ZORA-CCSD(T)/BS3+//ZORA-CCSD(T)/BS2+ using ORCA 5.0. All species correspond to minima (no imaginary frequencies).

$CH_3S^{\bullet}$

E = –275678.5161

S	-0.00044774	0.00898077	0.00000276
C	0.00138072	-1.79392052	0.00004146
H	-0.44346729	-2.19071990	-0.91062071
H	-0.49713990	-2.19067639	0.88248431
H	1.05127422	-2.09936396	0.03149217

$CH_3(O)S^{\bullet}$

E = –322896.7957

S	0.00011919	0.00499819	0.00125620
O	1.44164993	0.42391794	0.00921025
C	0.00117705	-1.80375965	0.00424125
H	0.51961621	-2.16048469	-0.88524270
H	0.50894881	-2.15751129	0.90103711
H	-1.03721120	-2.13816050	-0.00140212

$CH_3(O)_2S^{\bullet}$

E = –370104.2183

S	-0.00412648	0.00343255	-0.00000000
O	-0.57154077	0.42676629	1.27415684
O	-0.57154077	0.42676629	-1.27415684
C	0.00301329	-1.81001516	-0.00000000
H	-1.04355105	-2.11390610	-0.00000000
H	0.50987339	-2.13912294	0.90308556
H	0.50987339	-2.13912294	-0.90308556

$CH_3Se^{\bullet}$

E = –1559147.4317

Se	0.00021516	0.00069278	0.00002899
C	0.00639735	-1.94205147	-0.00301737
H	1.05507268	-2.24393349	0.01623053
H	-0.45668386	-2.32162715	-0.91005648
H	-0.49050133	-2.32448067	0.88471434

$CH_3(O)Se^{\bullet}$

E = –1606350.2613

Se	0.00028436	0.00001605	0.00000081
O	-1.61941599	0.35724069	-0.00000111
C	-0.00108320	-1.94800762	0.00000022
H	1.03297666	-2.29175046	-0.00000053
H	-0.52053143	-2.28524878	-0.89422749
H	-0.52053040	-2.28524988	0.89422810

$CH_3(O)_2Se^{\bullet}$

E = –1653528.0909

Se	-0.01096731	0.00180658	-0.00167516

O	-0.72850904	0.40659852	-1.40592709
O	-0.73657675	0.40418766	1.40599655
C	0.01449693	-1.95842693	0.00062683
H	-1.03259566	-2.25185442	0.00418593
H	0.53040306	-2.26816855	0.90491823
H	0.52354977	-2.26694286	-0.90812529
